# Galectin-3 induces pathogenic immunosuppressive macrophages through interaction with TREM2 in lung cancer

**DOI:** 10.1186/s13046-024-03124-6

**Published:** 2024-08-13

**Authors:** Qiaohua Wang, Yongjian Wu, Guanmin Jiang, Xi Huang

**Affiliations:** 1 Center for Infection and Immunity, Guangdong Provincial Engineering Research Center of Molecular Imaging, the Fifth Affiliated Hospital, Sun Yat-sen University, Zhuhai, China; 2 Department of Clinical Laboratory, the Fifth Affiliated Hospital, Sun Yat-sen University, Zhuhai, China; 3 Guangdong-Hong Kong-Macao University Joint Laboratory of Interventional Medicine, the Fifth Affiliated Hospital, Sun Yat-sen University, Zhuhai, China; 4 Zhuhai Engineering Research Center of Infection and Immunity, the Fifth Affiliated Hospital, Sun Yat-sen University, Zhuhai, China

**Keywords:** Galectin-3, Immunosuppression, Lung cancer, Macrophage, TREM2

## Abstract

**Background:**

High infiltration of tumor-associated macrophages (TAMs) is associated with tumor promotion and immunosuppression. The triggering receptor expressed on myeloid cells 2 (TREM2) is emerged as a key immunosuppressive regulator for TAMs, however, how TREM2-expressing TAMs are recruited and what ligands TREM2 interacts with to mediate immunosuppression is unknown.

**Methods:**

Flow cytometry and single-cell RNA sequencing were used to analyze TREM2 expression. Mechanistically, mass spectrometry and immunoprecipitation were employed to identify proteins binding to TREM2. Phagocytosis and co-culture experiments were used to explore the in vitro functions of galectin3-TREM2 pair. Establishment of *TREM2*^f/f^-Lyz2-cre mice to validate the role of TREM2 signaling pathway in lung carcinogenesis. GB1107 were further supplemented to validate the therapeutic effect of Galectin3 based on TREM2 signaling regulation.

**Results:**

This study identified that abundant TREM2^+^ macrophages were recruited at the intra-tumor site through the CCL2-CCR2 chemotactic axis. Galectin-3 impaired TREM2-mediated phagocytosis and promoted the conversion of TREM2^+^ macrophages to immunosuppressive TAMs with attenuated antigen presentation and co-stimulatory functions both in vitro both in vivo, and galectin-3 is a potential ligand for TREM2. Genetic and pharmacological blockade of TREM2 and galectin-3 significantly inhibited lung cancer progression in subcutaneous and orthotopic cancer models by remodeling the tumor immune microenvironment.

**Conclusion:**

Our findings revealed a previously unknown association between galectin-3 and TREM2 in TAMs of lung cancer, and suggested simultaneous inhibition of galectin3 and TREM2 as potent therapeutic approach for lung cancer therapy.

**Graphical Abstract:**

**Figure:**
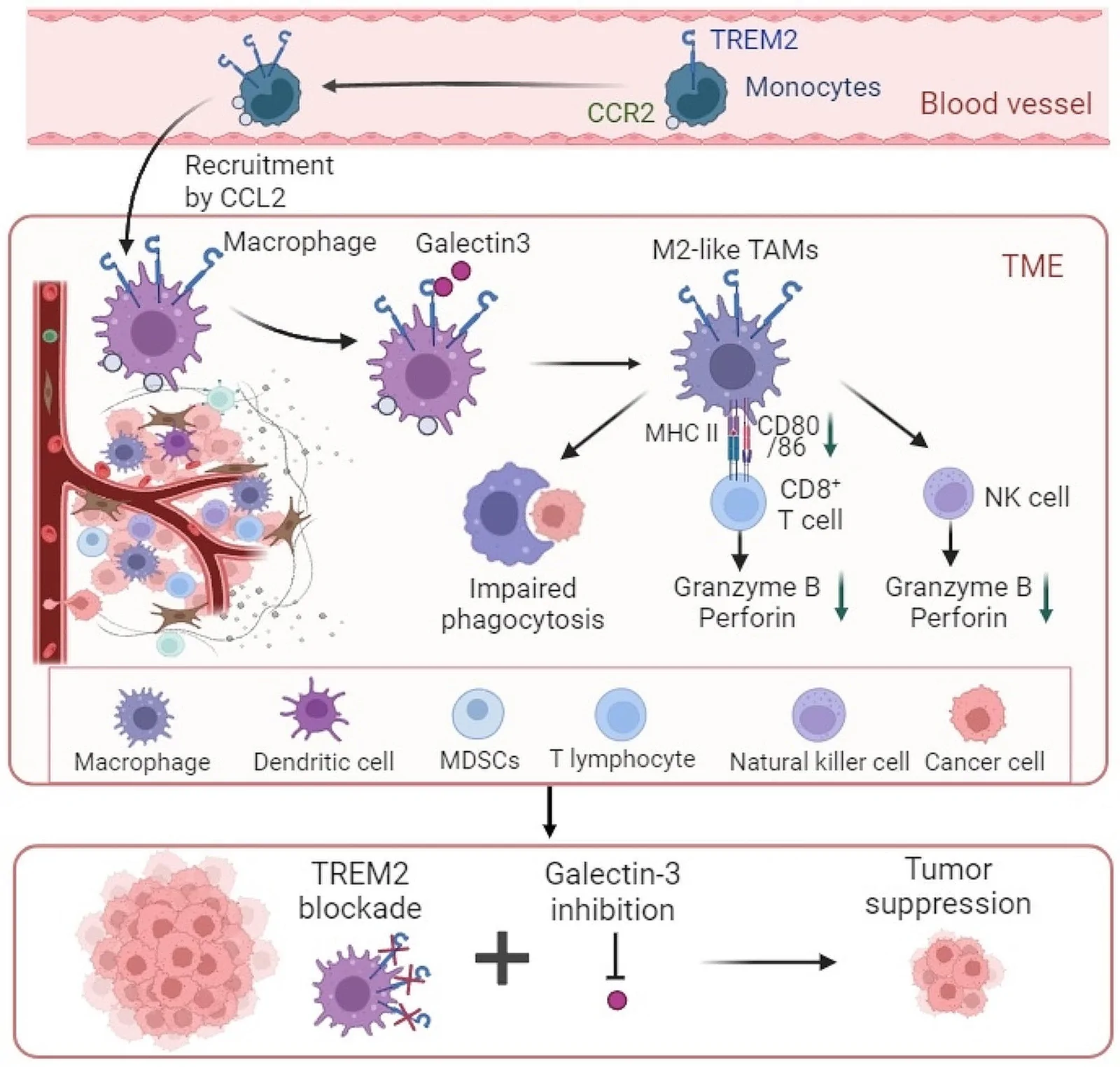

**Supplementary Information:**

The online version contains supplementary material available at https://doi.org/10.1186/s13046-024-03124-6.

## Background

The immune system is crucial for preventing tumorigenesis; however, tumor cells often induce an immunosuppressive tumor microenvironment (TME) that actively suppresses immune responses to evade immune surveillance [1]. For instance, cancer cells directly suppress T-cell responses by binding to adaptive immune checkpoints such as programmed death 1 (PD-1) [2] and cytotoxic T-lymphocyte-associated antigen 4 (CTLA-4) [3]. Immune checkpoint blockade (ICB) therapy has demonstrated efficiency in various cancers, including non-small cell lung cancer (NSCLC) [4], metastatic melanoma [5] and renal cancer [6]. However, owing to limitations such as the lack of tumor neoantigens [7], inability to reverse T cell exhaustion [8], and intratumoral presence of immunosuppressive immune cells, such as tumor-associated macrophages (TAMs) [9], only a small proportion of individuals respond to treatment. TAMs represent a significant innate immune cell population within the TME, accounting for approximately half of the local hematopoietic cells, with heterogeneity raging from anti- to pro-tumoral activity and differentiable plasticity [10]. The recruitment of TAMs from circulating monocytes/macrophages to the TME is primarily mediated by chemokines like CCL2, CCL5, and CXCL12 [11].

TAMs suppress the function of CD8^+^ tumor-infiltrating lymphocytes (TILs) and exert immunosuppressive effects by secreting interleukin-10 (IL-10) [12]. Clinically, increased intra-tumoral TAMs are associated with unfavorable outcomes across multiple solid tumor indications [13], indicating their significance as central mediators of immunosuppression in the TME. However, therapies that target TAMs reduction elicit poor anti-tumor responses. Therefore, novel strategies are required to precisely target TAMs and improve the efficacy and safety of TAMs-targeting therapies. The differentiation of TAMs to M2-like TAMs through complex cytokine connection inhibiting T-cell function and leading to tumor progression [14]. Current strategies against TAMs involve inhibiting their recruitment and differentiation, as well as re-educating TAMs to adopt an M1-like phenotype through activation or inhibition of checkpoint receptors. Among these, enhanced TAM-mediated phagocytosis plays a crucial role in tumor control by enhancing innate anti-tumor immunity and cross-activating T cell-mediated adaptive immune responses [15]. Therefore, identifying and targeting novel prophagocytic receptors will hold promise for advancing cancer immunotherapies against TAMs. We focused on a novel myeloid cell-expressed immunomodulator with prophagocytic activity, the triggering receptor expressed on myeloid cells 2 (TREM2). TREM2 is a transmembrane receptor belonging to the immunoglobulin superfamily, and has been recently identified in various tumors. TREM2 regulates phagocytosis, inflammation, metabolism, and cell survival by binding to the adaptor protein DNAX activation protein 12 (DAP12) [16]. The TREM2 receptor interacts with a variety of ligands, primarily anionic molecules, including bacterial products, DNA, and lipoproteins [17]. In addition, galectin-3 is identified as a potential endogenous ligand of TREM2 in Alzheimer’s disease (AD) [18]. Galectin-3 is a multifunctional protein of the beta-galactosidase-binding protein family [19]. Galectin-3 can promote macrophage differentiation to an M2-like phenotype and inhibit CD8^+^ T cell-mediated anti-tumor effects [20]. However, the phagocytic effects of TREM2 on tumor cells and the ligands that bind to TREM2 to exert specific phagocytic functions remain unclear.

TREM2 in TAMs fosters an immunosuppressive TME in different cancers [21]. Specifically, TREM2 in TAMs promotes tumor progression in colorectal carcinoma [22], ovarian carcinoma [23] and hepatocellular carcinoma (HCC) following transarterial chemoembolisation (TACE) [24] by remodeling the landscape of tumor-infiltrating myeloid cells and weakening the anti-tumor ability of CD8^+^ T cells. Additionally, TREM2 expression in cancer cells directly facilitates their survival, as observed in esophageal adenocarcinoma [25]. However, in gliomas, TREM2 inhibits tumor progression by enhancing the phagocytosis of tumor cells [26] and promoting MHC-II-related CD4^+^ T cell responses [27]. In summary, based on the different expression profiles and physiological functions of TREM2 and the heterogeneity of different cancer types, TREM2 exerts contrasting effects on tumor progression. In lung cancer, TREM2^+^ DCs exhibit immunosuppressive tumor-promoting properties [28], and TREM2^+^ mononuclear macrophages inhibit the accumulation and cytolytic activity of NK cells [29]. However, the upstream regulators of TREM2 in TAMs during the immune response to lung cancer need to be further elucidated.

Our findings indicated that abundant TREM2^+^ TAMs were recruited into the intratumor site through the CCL2-CCR2 chemotactic axis. In vitro, galectin-3 impairs TREM2-mediated phagocytosis and promotes the conversion of TREM2^+^ macrophages to pro-tumoral M2-like TAMs. Furthermore, liquid chromatography-mass spectrometry identified that soluble galectin-3 secreted by tumor cells was a potential ligand for TREM2. The galectin-3-TREM2 receptor signaling is responsible for TAMs infiltration and immunosuppressive. In vivo, combination therapy with TREM2 knockout and the galectin-3 inhibitor GB1107 substantially inhibited lung cancer progression. In summary, our results elucidated the galectin-3 is a potential ligand for TREM2 in TAMs. Notably, galectin-3-TREM2 may serve as a key immune checkpoint for tumor immune escape, providing a theoretical basis for providing potent targets against TAMs.

## Materials and methods

### Ethics statement and clinical samples

Between August 2019 to December 2020, 123 lung cancer patients (lung cancer group) from the outpatient department of the Fifth Affiliated Hospital of Sun Yat-sen University (Zhuhai, China) were included in this study. Inclusion and exclusion criteria: The clinical diagnosis of lung cancer in all included patients was based on chest imaging followed by tissue biopsy according to the Clinical Diagnosis and Treatment Guidelines for Lung Cancer of the Chinese Society of Oncology (2018 edition). Those with tissue biopsy results of non-malignant tumors were excluded. In addition, 64 healthy volunteers without clinical diseases were recruited from the Health Management Examination Centre of the Fifth Affiliated Hospital of Sun Yat-sen University. The research was approved by the Medical Ethics Committee of the Fifth Affiliated Hospital of Sun Yat-sen University (approval number: L233-1), and informed consent was obtained from all participants. Supplementary Table 1 provides comprehensive details regarding the clinical characteristics of these individuals.

### Mice

SPF-grade C57BL/6 wild-type (WT) mice (The Jackson Laboratory, RRID: IMSR_JAX: 000664) were procured from the Guangdong Provincial Laboratory Animal Centre. TREM2 KO mice (The Jackson Laboratory, RRID: IMSR_JAX: 027918), TREM2^f/f^ mice (mice carrying the loxP-flanked alleles of the TREM2 exon) (Nanjing GemPharmatech, RRID: N/A), Lyz2-cre mice (mice expressing Cre recombinase under the control of the Lyz2 promoter) (The Jackson Laboratory, RRID: IMSR_JAX: 032291), DAP12 KO mice (Nanjing GemPharmatech, RRID: N/A) and CD45.1 mice (The Jackson Laboratory, RRID: IMSR_JAX: 002014) were procured from GemPharmatech (Nanjing, China). All the mice were bred at the Laboratory Animal Centre of the Fifth Affiliated Hospital of Sun Yat-sen University. To generate myeloid-specific knockout mice for the TREM2 allele, breeding involved crossing TREM2^f/f^ mice with Lyz2-cre mice to achieve the lyz2-specific deletion of TREM2 (*TREM2*^f/f^-Lyz2-cre) mice. Mice were backcrossed to a C57BL/6 background for more than six generations. Genomic DNA extracted from tail biopsies was used for genotyping all mice via polymerase chain reaction (PCR). Male mice ranging from 6 to 8 weeks were employed for the experiments, with all animal procedures receiving approval from the Animal Ethics Committee of the Fifth Affiliated Hospital of Sun Yat-sen University (approval number: 00174). The tumor-bearing mice were assigned to each treatment group with 6–10 replications in randomization.

### Cells and transfection

The human lung cancer cell line A549 (ATCC, RRID: CVCL_4V07) and H292 (ATCC, RRID: CVCL_0455), mouse fibroblast cell line L929 (ATCC, RRID: CVCL_AR58), mouse lung adenocarcinoma cell line LLC (ATCC, RRID: CVCL_4358), mouse mononuclear macrophage leukemia cell line RAW264.7 (ATCC, RRID: CVCL_C6 × 3), and human embryonic kidney epithelial cell line 293T (ATCC, RRID: CVCL_0063) were procured from ATCC. These cell lines were cultured in Dulbecco’s modified Eagle’s medium (DMEM) (Gibco, Grand Island, USA) supplemented with 10% heat-inactivated fetal bovine serum (FBS) (Hyclone, Logan, USA), 100 U/mL penicillin, and 100 µg/mL streptomycin sulfate (pen-strep) (Invitrogen, California, USA) at 37 °C in a humidified atmosphere containing 5% CO2. Cell line authenticity was verified by STR analysis. LLC-luc-mCherry cells (luciferase-carrying LLC cells) were established in our laboratory with lenti-virus and cultured in DMEM. Plasmids and siRNAs were transfected into 293T cells and LLC using Lipofectamine 2000 (Invitrogen, California, USA), followed by cell harvesting 36 h after transfection. Mouse bone marrow derived macrophages (BMDMs) were generated following protocols. Briefly, after rinsing with phosphate-buffered saline (PBS), the femur and tibia were cultured in cell culture dishes supplemented with 30% (v/v) L929 conditioned medium (CM) as a source of colony-stimulating factor 1 (CSF-1). Replace the medium on day 4, and cells were used for phagocytosis assay on day 7. Mouse peritoneal macrophages were obtained as following protocols. Briefly, flushing the peritoneum with 5 mL of cold PBS for three times to isolate the peritoneal macrophages. The peritoneal macrophages will attach to the petri dishes. After 4 h, the non-macrophage cells were removed by washing with warm PBS for three times. Afterward, the adherent macrophages were cultured in complete RPMI 1640 supplemented with 30% (v/v) L929 CM. Replace the medium on day 4, and cells were used for phagocytosis assay on day 7. Peripheral blood mononuclear cells (PBMCs) were isolated using density gradient centrifugation with Ficol-Biocoll solution (MD Pacific, Tianjin, China). The PBMCs were seeded into cell culture dishes with serum-free RPMI 1640 medium and then incubated at 37 °C for 2 h. After gentle washing, the adherent cells, mainly representing monocytes, were cultured in RPMI 1640 medium supplemented with 10% human serum (Gimini, Beijing, China). Replace the medium on day 4, and cells were used for phagocytosis assay on day 7.

### In vitro phagocytosis assay

In a 24-well cell culture plate, 1 × 10^5^ macrophages were seeded overnight. The macrophages were then treated with the CM of tumor cells for 24 h, followed by incubation in a serum-free medium for 2 h. Subsequently, the target cells underwent washing and were labelled with 5 µM of carboxyfluorescein succinimidyl ester (CFSE) (CST, Boston, USA) or 2 µM of pHrodo iFL Green STP (Thermo Fisher Scientific, Waltham, Massachusetts, USA), and then 2 × 10^5^ CFSE-labelled target cells were introduced into the plate. After incubation at 37 °C for 2 h, the macrophages underwent extensive washing and were observed under an inverted microscope (OLYMPUS IX71, Tokyo, Japan). The phagocytic efficiency was determined by evaluating the number of macrophages that contained CFSE^+^ target cells per 100 macrophages. In the flow cytometry-mediated phagocytosis experiments, at the end of the phagocytic phase, all cells in the wells were collected and washed, and stained with anti-mouse F4/80 (BioLegend Cat# 123,116, RRID: AB_893481) or anti-human CD68 (BioLegend Cat# 333,810, RRID: AB_2275735) antibodies at 4 °C for 30 min, and then analyzed by flow cytometry (Attune NxT, Thermo Fisher Scientific, Waltham, USA). After gating the F4/80^+^ or CD68^+^ macrophages, the phagocytic efficiency was evaluated by measuring the percentage of F4/80^+^ or CD68^+^ cells that contained CFSE-derived green fluorescence in FlowJo (BD Biosciences, RRID: SCR_008520).

### Adoptive monocyte transfer assay

Adoptive monocyte transfer assay was performed as previously described [30]. Briefly, bone marrow from WT or *TREM2*^f/f^-Lyz2-cre mice was harvested, and monocytes were purified using Sony Flow cytometer (Sony, Tokyo, Japan). A total of 2 × 10^6^ monocytes were intravenously injected into the tail vein of CD45.1 recipient mice. After 24 h, a subcutaneous tumor transplantation assay was performed as previously described.

### Orthotopic lung tumor implantation assay

WT or *TREM2*^f/f^-Lyz2-cre mice aged 6–8 weeks were anesthetized with 2% isoflurane (RWD, Shenzhen, China). A total of 30 µL of PBS or matrigel (Corning, New York, USA) containing 1 × 10^6^ luciferase-carrying LLC (LLC-luc) cells was injected into the left lung parenchyma from 1 cm above the lower rib line. The incisions were sealed using absorbable sutures. During and after the surgery, the mice were kept on a heating pad until they regained consciousness from anesthesia. On day 19, the mice were euthanized, and the lungs were collected for tumor counting and HE staining. In the survival studies (*n* = 10), each mouse was injected with 5 × 10^6^ LLC-luc cells in the lung to obtain the desired survival time.

### Co-immunoprecipitations (CO-IP) and immunoblots

For exogenous CO-IP, 293T cells were transfected with HA/Flag tagged plasmids, and the cell lysate was incubated with anti-HA agarose beads or anti-Flag M2 affinity beads overnight at 4 ℃. For endogenous CO-IP, tumor-infiltrating macrophages were lysed and incubated with anti-TREM2 (Cell Signaling Technology Cat# 91,068, RRID: AB_2721119) and protein A/G plus-agarose (Merck Millipore, Massachusetts, USA) overnight at 4 ℃. Immunoblots were performed as following protocols. Briefly, cell lysate was lysed in RIPA lysis (Ding guo, Guangzhou, China) for 30 min at 4 ℃, subsequently, it was centrifuged and boiled after the addition of loading buffer. After running and transferring the protein to PVDF membrane (Roche, Basel, Switzerland), the membrane underwent sequential incubation with primary and secondary antibodies, and then the PVDF membrane was observed using with LAS500 ultrasensitive chemiluminescence imager (ImageQuant, RRID: SCR_014246). The grey value of the protein band was analyzed using the ImageJ gel image analyses software, and the relative grey value was normalized to the grey value of β-actin. Image J (Fiji, RRID: SCR_002285) was used to analyze the grey value of each protein band, and the grey value of β-actin was used as a standard value.

### Real-time fluorescence quantitative PCR (RT-qPCR)

After extracting total RNA using TRIZOL (Invitrogen, CA, USA), 1 µg of total RNA was reversely transcribed into cDNA. Then the mixture of cDNA, specific primers listed in Supplementary Tables 2, and SYBR green (Applied Biosystems, CA, USA) were amplified using the CFX96 fluorescence quantitative PCR instrument (Bio Rad, CA, USA). β-actin was used to normalize the relative gene expression, and the ΔΔCt method was used to calculate the fold change in mRNA expression.

### Liquid chromatography-mass spectrometry

Following the surgical procedure, human lung cancer tissues were finely minced using razor blades on ice. The harvested cells were lysed using RIPA lysis buffer and subjected to immunoprecipitation with either anti-TREM2 antibody (Cell Signaling Technology Cat# 91,068, RRID: AB_2721119) or control IgG along with protein A/G agarose beads (Merck Millipore, Massachusetts, USA) overnight at 4 °C. The unbound proteins were washed away, and the agarose beads bound to the antibodies and the corresponding proteins were collected for analyses. Then the liquid chromatography–mass spectrometry was performed on an Orbitrap Fusion Lumos (ThermoFisher Scientific, Waltham, USA).

### Reagents

**Flow cytometry analyses** involved the following antibodies: PE-anti-human/mouse TREM2 (R&D Systems Cat# FAB17291P, RRID: AB_884528) was from R&D Systems (Minnesota, USA). PE-anti-human/mouse Galectin-3 (BioLegend Cat# 125,405, RRID: AB_2136764), FITC-anti-mouse CD45.2 (BioLegend Cat# 109,806, RRID: AB_313443), FITC-anti-mouse CD11b (BioLegend Cat# 101,206; RRID: AB_312788), APC-anti-mouse F4/80 (BioLegend Cat# 123,116, RRID: AB_893481), PerCP-Cy5.5-anti-mouse CD3 (BioLegend Cat# 100,218, RRID: AB_1595492), APC/Cyanine7-anti-human CD45 (BioLegend Cat# 982,310, RRID: AB_2715773), APC anti-human CD68 (BioLegend Cat# 333,810, RRID: AB_2275735), PE-anti-mouse CD45.1 (BioLegend Cat# 110,707, RRID: AB_313496), APC/Cyanine7-anti-human/mouse Ly6G/Ly6C (Gr1) (BioLegend Cat# 108,423, RRID: AB_2137486), PE-anti-mouse Granzyme B (BioLegend Cat# 372,208, RRID: AB_2687032), APC/Cyanine7-anti-mouse CD8 (BioLegend Cat# 128,011, RRID: AB_1659242), PerCP-Cy5.5-anti mouse-Ly6C (BioLegend Cat# 109,806, RRID: AB_313443), FITC-anti-human CD4 (BioLegend Cat# 317,408, RRID: AB_571951), PerCP-Cy5.5-anti-mouse NK1.1 (BioLegend Cat# 108,728, RRID: AB_2132705), FITC-anti-mouse CD3 (BioLegend Cat# 100,204, RRID: AB_312661), PE/Cyanine7-anti-mouse CD206 (MMR) (BioLegend Cat# 141,720, RRID: AB_2562248), PE-anti-Nos2 (iNOS) (BioLegend Cat# 696,805, RRID: AB_2876745), APC-anti-mouse H-2K^b^ (MHC I) (BioLegend Cat# 116,518, RRID: AB_10564404), PE-anti-mouse I-A/I-E (MHC II) (BioLegend Cat# 107,607, RRID: AB_313322), PE-anti-mouse CD86 (BioLegend Cat# 159,204, RRID: AB_2832568), APC-anti-mouse CD80 (BioLegend Cat# 104,714, RRID: AB_313135), and APC-anti-mouse CCR2 (BioLegend Cat# 150,627, RRID: AB_2810414), APC-anti-mouse Perforin (BioLegend Cat# 154,303, RRID: AB_2721462), FITC-anti-mouse CD4 (BioLegend Cat# 100,510, RRID: AB_312713), FITC-anti-human CD3 (BioLegend Cat# 300,406, RRID: AB_314060) were obtained from Biolegend (California, USA). Fixable Viability Dye eFluor™ 506 (Thermo Fisher Scientific Cat# 65-0866-14) was from Thermo Fisher Scientific (Waltham, USA).

**Immunoprecipitations**,** immunoblots and Immunofluorescence** involved the following antibodies: Anti-Flag-tag (DYKDDDK) (Cell Signaling Technology Cat# 14,793, RRID: AB_2572291), anti-Myc-tag (Cell Signaling Technology Cat# 2276 S, RRID: AB_331783), anti-HA-tag (Cell Signaling Technology Cat# 3724 S, RRID: AB_1549585), anti-Syk (Cell Signaling Technology Cat# 2712, RRID: AB_2197223), anti-Src (Cell Signaling Technology Cat# 2109, RRID: AB_2106059), anti-β-actin (Sigma-Aldrich Cat# A5316, RRID: AB_476743), anti-Galectin-3 (R and D Systems Cat# MAB1197, RRID: AB_2136769), anti-Phospho-Syk (Tyr525/526) (Cell Signaling Technology Cat# 2710, RRID: AB_2197222), anti-Phospho-Src (Tyr419) (Affinity Biosciences Cat# AF3162, RRID: AB_2834597), Anti-TREM2 (D8I4C) (Cell Signaling Technology Cat# 91,068, RRID: AB_2721119), Anti-TREM2 (R&D Systems Cat# AF1828, RRID: AB_2208689), and Anti-CD68 (EPR20545) (Abcam Cat# ab213363, RRID: AB_2801637).

#### Agonists and inhibitors

Tyrosine kinase inhibitor (Genistein) (absin, Shanghai, China), Syk inhibitor (R406) (Selleck Chemicals, Houston, USA), Src inhibitor (PP2) (Solarbio, Beijing, China), TREM2 Fc (R&D Systems, Minnesota, USA), GB1107 (Selective Galectin-3 inhibitor) (TargetMol, Boston, Massachusetts), Recombinant mouse Galectin-3 protein (rm Galectin-3) (R&D Systems, Minnesota, USA).

#### ELISA kits

Human GAL3 (Galectin3) ELISA Kit was from Elabscience (Wuhan, China). Mouse CCL2/MCP1 ELISA Kit was from MEIMIAN (Jiangsu, China). Mouse Granzyme B ELISA Kit and Mouse Perforin ELISA kit were from Abcam (Cambridge, England).

### CM collection

When the LLC, A549 or L929 cells reached approximately 50% confluence, the medium was changed to fresh complete medium. Two days later, the CM was centrifuged at 4 °C for 5 min, 10 min, and 20 min at 500 g, 2000 g, and 2000 g, respectively. The medium was then stored at -80 ℃.

### Immunofluorescence (IF) and immunohistochemistry (IHC)

293T cells overexpressing TREM2-HA and Galectin-3-Flag were washed and fixed with 4% paraformaldehyde (BBI Life Sciences, Shanghai, China), then permeabilized and blocked with 3% Triton X-100- and 5% BSA-containing PBS for 1 h. Following overnight incubation with anti-HA (Cell Signaling Technology Cat# 3724 S, RRID: AB_1549585) and anti-Flag (Cell Signaling Technology Cat# 14,793, RRID: AB_2572291) primary antibodies, the cells were subsequently stained with Alexa Fluor 488-conjugated goat anti-mouse IgG (Invitrogen Cat# A-11,059, RRID: AB_2534106) and Alexa Fluor 594-conjugated goat anti-rabbit IgG (Abcam Cat# ab150080, RRID: AB_2650602) for 1 h. In the case of paraffin sections, anti-CD68 (EPR20545) (Abcam Cat# ab213363, RRID: AB_2801637) and anti-TREM2 (R&D Systems Cat# AF1828, RRID: AB_2208689) antibodies were applied. Following this, cell nuclei were counterstained with DAPI (Invitrogen, California, USA) for 5 min and observed using an LSM 780 laser-scanning confocal microscope (Carl Zeiss, Oberkochen, Germany). **For immunohistochemistry experiments**, after secondary antibodies incubation, the solution containing streptomyces complex antitoxin peroxidase was applied and followed by an 1 h-incubation at room temperature. Finally, the DAB staining (Boster, Wuhan, China) was performed, and visualized using the inverted fluorescence microscope. **For actin polarization experiments**, 2 × 10^5^ RAW264.7 cells were seeded in the confocal dishes. The next day, cells were stimulated with LLC CM along with GB1107 (5 µM) for 24 h. After mixing macrophages and CFSE-labelled LLC at a ratio of 1:2 and following an incubation at 37 °C for 30 min, the cells then underwent fixation, washing, permeabilization, and blocking, similar to 293T cells as detailed above. Subsequently, an incubation with anti-β-actin antibody (AC-74) (Sigma-Aldrich Cat# A5316, RRID: AB_476743) was performed overnight at 4 °C, followed by an incubation with Alexa Fluor 488-conjugated goat anti-mouse IgG (Invitrogen Cat# A-11,059, RRID: AB_2534106) for 1 h. Then the cells were observed using an LSM 780 laser-scanning confocal microscope.

### Hematoxylin-eosin (H&E) staining

Following euthanasia of the mice, the lungs were harvested and fixed overnight in 4% paraformaldehyde. Subsequently, the lungs were embedded in paraffin and sliced into 5 μm sections. H&E staining (Biosharp, Beijing, China) was conducted, and the samples were analyzed using an optical microscope (Olympus FV1000, Tokyo, Japan).

### Intraperitoneal tumor clearance assay

WT or *TREM2*^f/f^-Lyz2-cre mice received an intraperitoneal injection of 200 µL of LLC CM for 2 days, and then the mice were intraperitoneally injected with 5 × 10^6^ CFSE-labelled LLC in 100 µL of PBS. After 24 h, the cells presented in the peritoneal cavity were harvested with 2% FBS-containing PBS, and the remaining CFSE^+^ target cells were quantified using flow cytometry.

### Subcutaneous tumor transplantation assay

WT, *TREM2*^f/f^-Lyz2-cre, or DAP12 KO mice were subcutaneously injected with approximately 1 × 10^6^ LLC into the right flank. The tumor volume was measured on alternate days using a caliper and calculated using the following formula: (length × width^2^)/2. Starting on day 5, the galectin-3 inhibitor, GB1107, was administered orally at a dose of 10 mg/kg daily as previously described [20]. The experiment was concluded before the tumors reached the allowable size limit of 1.5 cm in diameter. Following the termination of the experiment, the tumors were dissected, weighed, sectioned into small slices, filtered over a 40-µm filter, and washed with PBS. The tumor-infiltrating immune cells were then stained with relevant antibodies and detected using flow cytometry. Additionally, the tumor grinding supernatant was analyzed by ELISA.

### Whole-body bioluminescence imaging

As previously described [31], the D-luciferin potassium salt (Abmole, shanghai, China) was reconstituted in PBS and intraperitoneally injected to tumor-bearing mice at a dosage of 150 mg/kg. After being anesthetized with 2% isoflurane, the mice underwent imaging with the Caliper IVIS Lumina III system (Perkin Elmer, Waltham, Massachusetts, USA) 15 min following the injection of D-luciferin potassium salt. The Region of Interest (ROI) was specified as a circle with a radius of 2 cm covering the lung area. Total flux (photos/s) and average radiance (p/s/cm^2^/sr) within the ROI were measured using the Caliper Life Sciences Living Image software.

### Solid-phase binding assay

The experimental procedures of solid-binding assay were previously described [32]. Following overnight coating of the 96-well plate with TREM2-Fc or control IgG (2 µg/mL) (R&D Systems, Minneapolis, USA) in PBS overnight at 4 °C, the plate was washed and subsequently blocked with 3% BSA in PBS for at 37 °C 1 h. Next, the recombinant human galectin-3 protein (R&D Systems, Minneapolis, USA) was diluted and added to the plate at indicated concentrations in PBS containing 0.5% BSA, followed by an incubation at 37 °C for 1 h. Subsequently, the binding of galectin-3 protein was detected using biotinylated anti-galectin-3 antibody (Elabscience, Cat# E-AB-22,006) at 37 °C for 1 h. Then the plate underwent washing and subsequent incubation with avidin–horseradish peroxidase (Elabscience, Wuhan, China) at 37 °C for 30 min. Following another round of washing, the plate was exposed to the TMB substrate solution (Elabscience, Wuhan, China), and the absorbance was measured at 450 nm.

### Statistical analyses

GraphPad Prism (San Diego, RRID: SCR_002798) was used for the statistical analyses, with data presented as mean ± SD. *P* values were calculated using a two-tailed Student’s t-test and one-way or two-way analyses of variance (ANOVA), for calculating the comparison of one group and more than one group, respectively. For survival analyses, *P* values were calculated using the log-rank (Mantel–Cox) test. A *P* < 0.05 was considered statistically significant.

## Results

### TREM2^+^macrophages are increased and recruited in the intratumor site of lung cancer via the CCL2-CCR2 axis

**Fig. 1:**
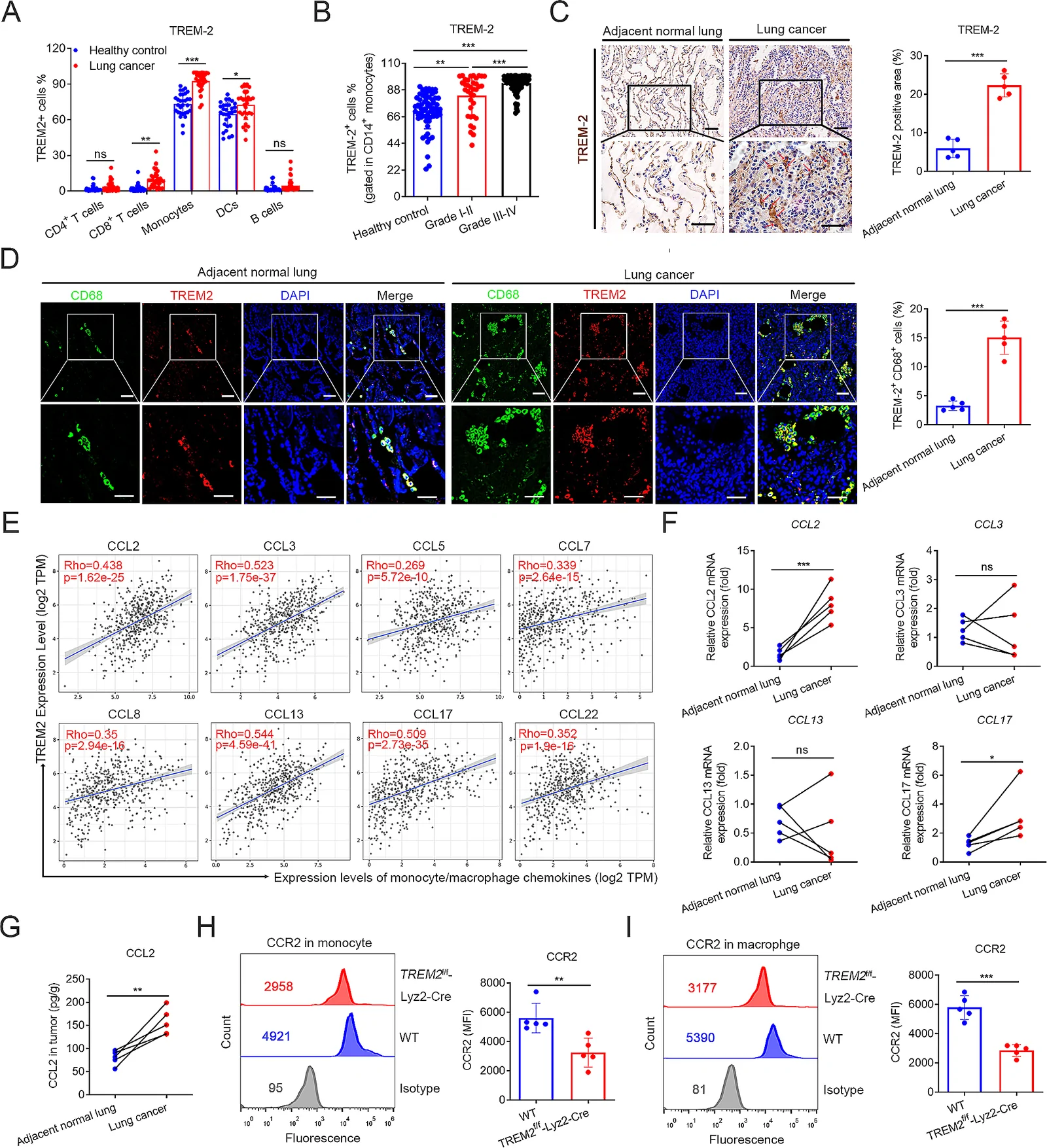
TREM2^**+ **^macrophages are increased and recruited in the intratumor site of lung cancer via the CCL2-CCR2 axis. (**A**) Proportion of TREM2 expression in PBMCs from lung cancer patients (*n* = 35) and healthy donors (*n* = 30) were analyzed by flow cytometry. (**B**) Proportion of TREM2 expression in CD14^+^ monocytes from healthy donors (*n* = 64) and lung cancer patients (*n* = 123) with different TNM stages was analyzed by flow cytometry. (**C**) Immunohistochemical staining of TREM2 in adjacent normal lung and intratumor lung tissues (*n* = 5). Scale bars, 20 μm. (**D**) Immunofluorescence staining of TREM2^+^ TAM in adjacent normal lung and intratumor lung tissues (*n* = 5) by DAPI counterstaining. Scale bars, 20 μm. (**E**) Correlation analyses between TREM2 expression abundance and various monocyte chemokines of lung cancer in the TCGA database. (**F**) Main monocyte chemokine levels in lung cancer and the adjacent normal lung were determined by RT-qPCR. (**G**) Monocyte chemokine CCL2 levels in lung cancer and the adjacent normal lung were determined by ELISA. (**H-I**) CCR2 expression in peripheral blood monocytes (**H**) and tumor infiltrating macrophages (**I**) of lung cancer-bearing mice were determined by flow cytometry. Data represent mean ± SD from three experiments. *, *P* < 0.05; **, *P* < 0.01; ***, *P* < 0.001. ns, no significance

TREM2 is identified as a key player in the regulation of tumor-associated myeloid cells [21], however, its specific role and immune regulatory mechanisms in lung cancer remains unclear. To evaluate the expression profile of TREM2 in lung cancer, we initially analyzed peripheral blood mononuclear cells (PBMCs) of 35 lung cancer patients and 30 healthy volunteers. Consistent with previous results, TREM2 expression was higher in monocytes of lung cancer [28] (Fig. 1A and Supplementary Fig. 1A). Further analyses between the TNM stage of lung cancer and TREM2 expression indicated increased TREM2 expression in the monocytes of patients with both early and advanced lung cancer. Moreover, there was a higher expression of TREM2 observed in patients with advanced lung cancer compared to those with early-stage disease (Fig. 1B and Supplementary Fig. 1B). In the TME of lung cancer, immunohistochemical staining revealed elevated expression of TREM2 in intratumor tissues, resembling tumor-infiltrating macrophages (Fig. 1C). The correlation analyses between TREM2 and TILs in lung cancer performed using the TIMER 2.0 website (cistrome.org) [33], showed that TREM2 expression exhibited a strong correlation with mononuclear macrophages (Supplementary Fig. 1C). Moreover, the immunofluorescence results showed a clear and abundant population of cells co-expressing CD68 and TREM2 in human lung cancer than that in healthy lung (Fig. 1D). Similarly, in mouse lung cancer models, TREM2 expression was upregulated in both lung-infiltrating macrophages and splenic monocytes in tumor-bearing mice, which is consistent with observations in humans (Supplementary Fig. 1D).

TAMs are usually dependent on chemokines such as CCL2, CCL5 and CXCL12 for recruitment from peripheral monocytes/macrophages into the TME at the primary tumor site [11]. To investigate the relationship between TREM2^+^ macrophages and circulating monocytes, we analyzed the correlation between TREM2 and major monocyte chemokines through the TIMER2.0 website (cistrome.org) [33]. We found that TREM2 was strongly associated with CCL2, CCL3, CCL13, and CCL17 in lung cancer (Fig. 1E). RT-qPCR analyses of CCL2, CCL3, CCL13, and CCL17 revealed that the transcription level of CCL2 in lung cancer was notably elevated compared to that in healthy lung (Fig. 1F). Moreover, the protein level of CCL2 was elevated in lung cancer (Fig. 1G). In the TME, monocytes/macrophages often express CCR2 and bind to CCL2 to form a chemotactic axis for recruitment as TAMs [34]. Our examination of CCR2 expression in monocytes/macrophages indicated a reduction in TREM2 knockout monocytes/macrophages (Fig. 1H and I). Collectively, these findings confirmed that the CCL2-CCR2 chemotactic axis may contribute to the increase in TREM2^+^ macrophages in lung cancer.

### Tumor educated TREM2^+^ macrophages have impaired phagocytosis activity and acquire an M2-like phenotype

For an exploration of the up-regulated TREM2 in macrophages, we analyzed the single cell RNA sequencing data of human lung adenocarcinoma [35], and identified TREM2-positive macrophages (TREM2^+^ M) and TREM2-negative macrophages (TREM2^−^ M) (Supplementary Fig. 2A-C). Furthermore, GO-BP analyses revealed that signaling pathways associated with phagocytosis, antigen presentation and activation of T cells, which are closely related to phagocytosis [36], were enriched in TREM2^+^ macrophages (Supplementary Fig. 2D), suggesting that the upregulation of TREM2 in mononuclear macrophages may be associated with altered phagocytosis.

**Fig. 2:**
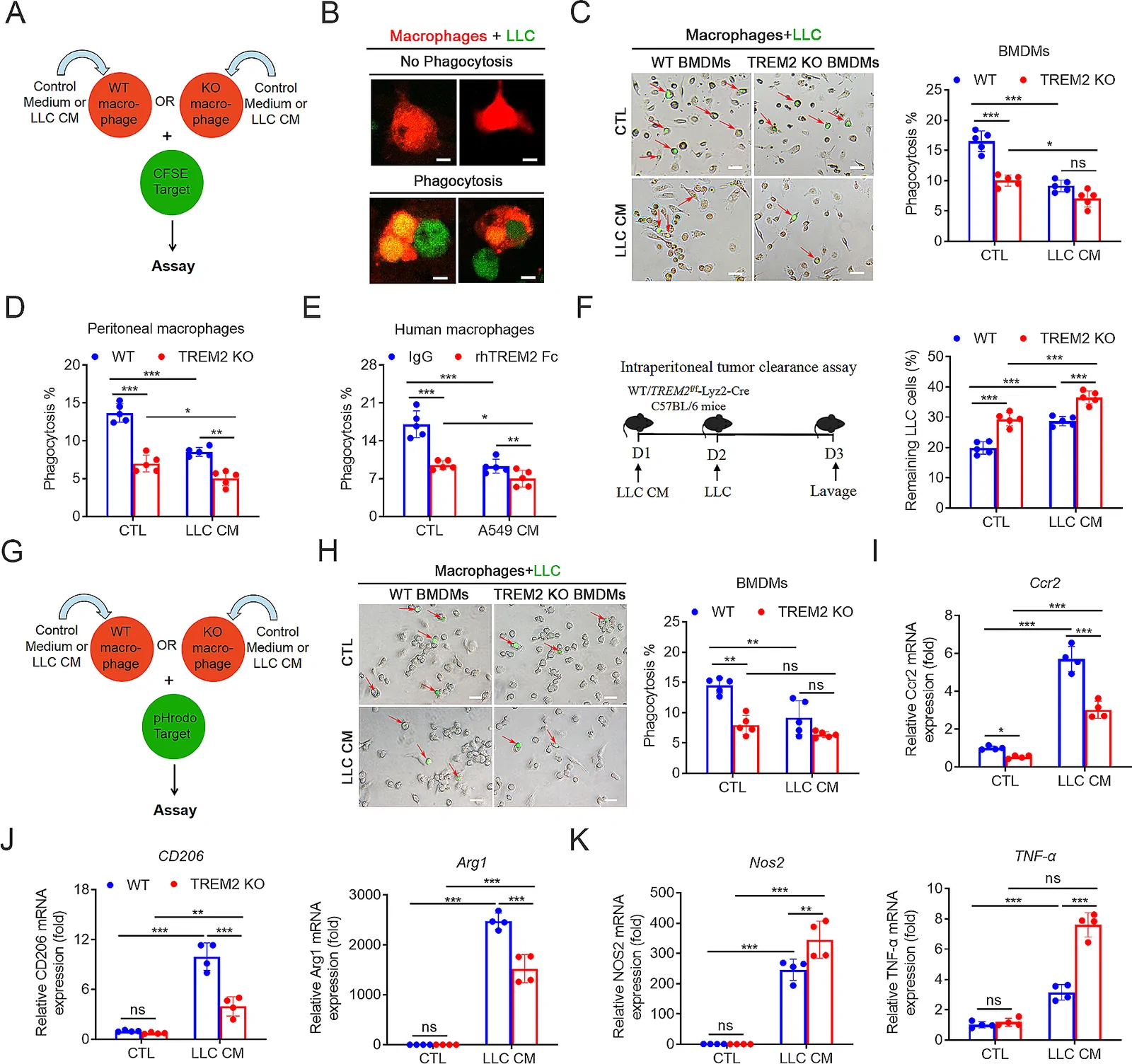
Tumor-educated TREM2^**+ **^macrophage has impaired phagocytosis activity and acquires an M2-like phenotype. (**A**) Flowchart of phagocytosis experiment. (**B**) Demonstration of phagocytic morphology of macrophages on tumor cells. Macrophages, red; tumor cell, green. Scale bars, 5 μm. (**C**) Phagocytosis (red arrows) of tumor cells (green) by LLC CM-pretreated WT or TREM2 KO BMDMs with fluorescence microscopy. Scale bars, 50 μm. (**D**) Phagocytosis assay between WT or TREM2 KO peritoneal macrophages which were pretreated with LLC CM for 24 h and LLC were detected by flow cytometry. (**E**) Phagocytosis assay between A549 CM-pretreated human macrophages which were then blocked with rhTREM2 Fc (1 µg/mL) and LLC was detected by flow cytometry. (**F**) Intraperitoneal tumor cell clearance experiments between LLC CM-pretreated WT or TREM2 KO mice and LLC. (**G**) Flow chart of phagocytosis assay using pHrodo green dye. (**H**) Phagocytosis (red arrows) of pHrodo green labelled LLC (green) by LLC CM-pretreated WT or TREM2 KO BMDMs with fluorescence microscopy. Scale bars, 50 μm. (**I-K**) After 24 h of LLC CM treatment, the transcription levels of *Ccr2*(**I**), M2-like macrophage markers (*CD206*, *Arg1*) (**J**) and M1-like macrophage markers (*Nos2*, *TNFα*) (**K**) in WT or TREM2 KO BMDMs were detected by RT-qPCR. Data represent mean ± SD from three experiments. *, *P* < 0.05; **, *P* < 0.01; ***, *P* < 0.001. ns, no significance

To further explore whether TREM2 affects macrophage-mediated phagocytosis, bone marrow-derived macrophages from WT mice (WT BMDMs) and *TREM2*^f/f^-Lyz2-Cre mice (TREM2 KO BMDMs) were isolated and cultured for phagocytosis experiments (Fig. 2A). Immunofluorescence images showed that the macrophages were able to phagocytose LLC (Fig. 2B), and phagocytosis rate of TREM2 KO BMDMs was attenuated (Fig. 2C). In addition, to investigate phagocytosis in tumors, BMDMs were stimulated with 50% tumor cell-conditioned medium derived from LLC (LLC CM) in vitro. Surprisingly, LLC CM significantly reduced the phagocytic activity of WT BMDMs similar to that of TREM2 KO BMDMs (Fig. 2C), indicating that the tumor cell-CM may have transformed the phenotype of the macrophages, resulting in a reduction in macrophage function, including phagocytosis. Similarly, a phagocytosis assay between mouse peritoneal macrophages or human monocyte-derived macrophages and tumor cells showed that TREM2 deficiency/blockade or tumor cell-CM treatment reduced the phagocytosis rate (Fig. 2D and E). Furthermore, the results of in vivo intraperitoneal tumor cell clearance assay showed that *TREM2*^f/f^-Lyz2-Cre mice exhibited reduced ability to clear tumor cells (Fig. 2F). The pHrodo IFL green STP ester, a novel pH-responsive fluorescent dye for phagocytosis, was employed to validate the impact of TREM2 deficiency or LLC CM on the phagocytic activity of macrophages (Fig. 2G and H). Immunosuppressive M2-like macrophages have reduced phagocytic capacity [37]. Therefore, tumor cell-CM may down-regulate phagocytic function by converting macrophages into an M2-like phenotype. The LLC CM-stimulated BMDMs were analyzed for macrophage differentiation-related genes and TREM2 deficiency notably decreased M2-like phenotypic markers (*CD206* and *Arg1*) expression (Fig. 2J), and critically increased M1-like phenotypic markers (*Nos2* and *TNFα*) expression (Fig. 2K) in LLC CM-treated macrophages. In addition, LLC CM increased transcription level of *Ccr2*, which was inhibited by TREM2 deficiency (Fig. 2I). These results suggested that tumor cell-CM impairs macrophage-mediated phagocytosis and converts macrophages to an M2-like phenotype which depends on TREM2.

### Tumor derived galectin-3 is a ligand for TREM2

**Fig. 3:**
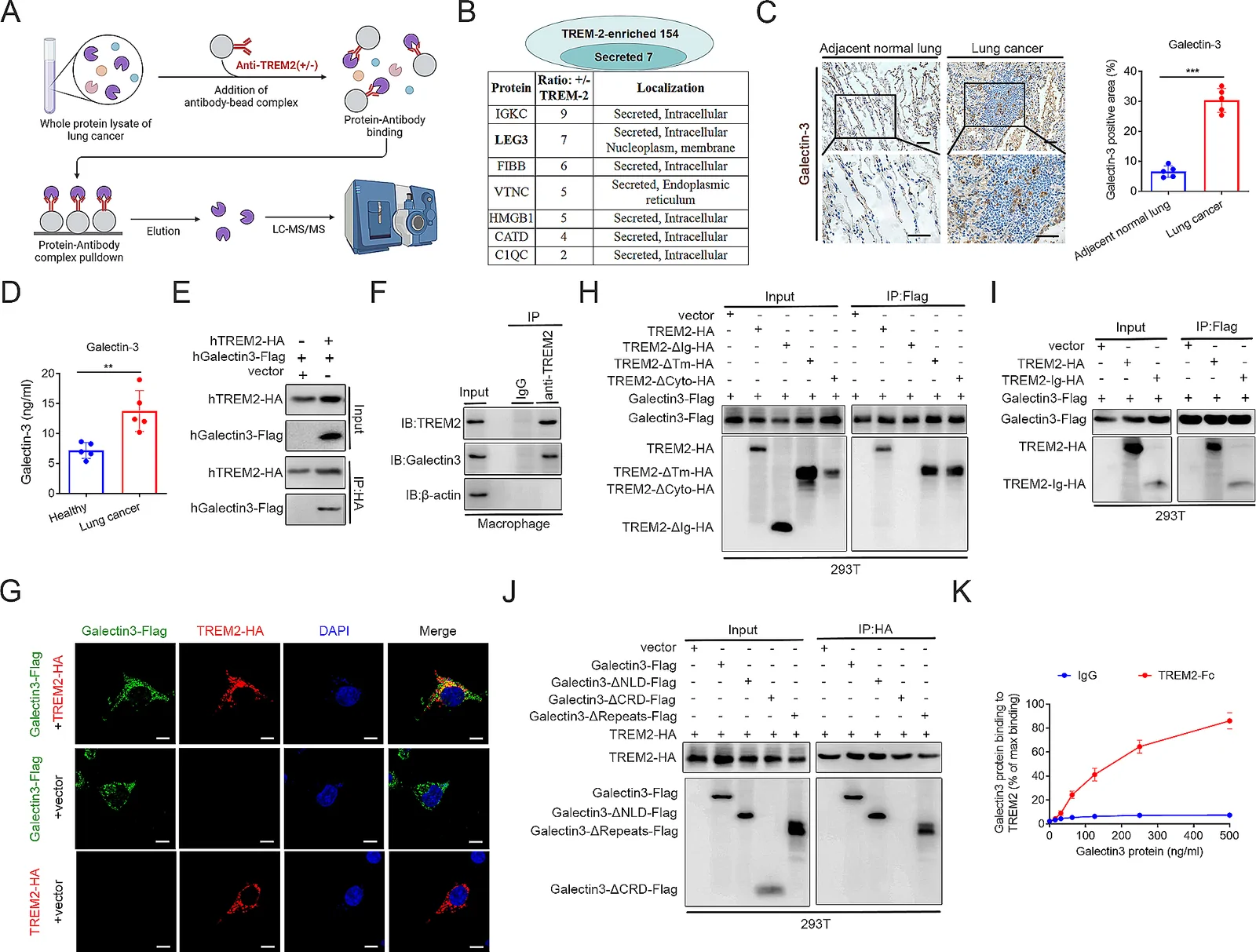
Tumor derived galectin-3 is a ligand for TREM2. (**A**) Illustration of human lung cancer tissue protein precipitated by anti-TREM2 or IgG antibody (*n* = 3), and peptides enriched in each complex were identified by mass spectrometry. (**B**) Venn diagram and tables showing secreted proteins concentrated in the TREM2 enriched complex. (**C**) Immunohistochemical staining of galectin-3 in adjacent normal lung and intratumor areas of lung tissues (*n* = 5). Scale bars, 20 μm. (**D**) Galactin-3 levels in lung cancer or normal lung lavage were determined by ELISA. (**E**) 293T cells were transfected with pcDNA3.1-vector/pcDNA3.1-hTREM2-HA/pcDNA3.1-hGalectin-3-Flag plasmids as indicated. Anti-HA antibody was employed for exogenous CO-IP experiments. (**F**) F4/80^+^ macrophages were sorted from human lung cancer tissue. Anti-TREM2 antibody was employed for endogenous CO-IP experiment. (**G**) 293T cells were transfected with pcDNA3.1-TREM2-HA and pcDNA3.1-Galectin-3-Flag plasmids. The co-localization between TREM2 and Galectin-3 was examined using confocal microscopy. Scale bars, 5 μm. (**H**) 293T cells were transfected with pcDNA3.1-TREM2-HA/pcDNA3.1-TREM2-ΔIg-HA/pcDNA3.1-TREM2-ΔTm-HA/pcDNA3.1-TREM2-ΔCyto-HA/pcDNA3.1-Galectin-3-Flag/pcDNA3.1-vector plasmids as indicated. Anti-Flag antibody was employed for exogenous CO-IP experiments. (**I**) 293T cells were transfected with pcDNA3.1-vector/pcDNA3.1-TREM2-Ig-HA/pcDNA3.1-TREM2- HA/pcDNA3.1-Galectin-3-Flag plasmids as shown. Anti-Flag antibody was employed for exogenous CO-IP experiments. (**J**) 293T cells were transfected with pcDNA3.1-vector/pcDNA3.1-TREM2-HA/pcDNA3.1-Galectin-3-ΔNH2-Flag/pcDNA3.1-Galectin-3-ΔCRD-Flag/pcDNA3.1-Galectin-3- ΔRepeats-Flag plasmids as indicated. Anti-HA antibody was employed for exogenous CO-IP experiments. (**K**) Galectin-3 protein bound to TREM2 in the plate was determined using anti-Galectin-3 antibody. Data represent mean ± SD from three experiments. **, *P* < 0.01; ***, *P* < 0.001

To elucidate how tumor cell-CM modulates TREM2-mediated phagocytosis and phenotypic transformation of macrophages, immunoprecipitation and mass spectrometry analyses were performed (Fig. 3A). Liquid chromatography-mass spectrometry identified 154 proteins that were more than 2-fold enriched in the anti-TREM2 group, and 7 of the 154 proteins were secreted proteins (Fig. 3B and Supplementary Table 3). Among these secreted proteins, galectin-3 exhibits high expression levels in non-small cell lung cancer (NSCLC), and galectin-3 deficiency or pharmacological blockade significantly inhibits lung cancer progression [20]. In addition, galectin-3 serves as an intrinsic ligand for TREM2 in AD-associated microglia [18]. Immunohistochemical analyses demonstrated a high expression of galectin-3 in lung cancer (Fig. 3C). ELISA results demonstrated a significant increase in the protein level of galectin-3 in lung cancer compared to normal lung (Fig. 3D), indicating that galectin-3 may have a crucial regulatory function in the progression of lung cancer. To investigate the relationship between TREM2 and galectin-3, co-immunoprecipitation (CO-IP) experiments were performed. The exogenous CO-IP results showed that both human- and mouse-derived TREM2 and galectin-3 could bind to each other (Fig. 3E and Supplementary Fig. 3A-C). The interaction between TREM2 and galectin-3 was also confirmed in F4/80^+^ macrophages from patients with lung cancer using endogenous CO-IP experiments (Fig. 3F). Moreover, immunofluorescence experiments showed obvious co-localization of TREM2 and galectin-3 in 293T cells (Fig. 3G). To further explore the specific binding domains of TREM2 and galectin-3, exogenous CO-IP experiments were performed, which demonstrated that TREM2 full-length, TREM2-Ig (immunoglobulin-like) domain alone, and TREM2-lacking either the transmembrane (∆TM) or the cytoplasmic (∆Cyto) domain could all interact with galectin-3; however, this binding disappeared in the absence of the Ig domain (∆Ig) (Fig. 3H and I). In addition, galectin-3 lacking either the N-terminal (∆NLD) or tandem repeat sequence (∆Repeats) domain could interact with TREM2; however, this binding disappeared in the absence of carbohydrate recognition domain (∆CRD) (Fig. 3J), indicating that Galectin-3 combined with TREM2 via its carbohydrate recognition domain (CRD). To validate the interaction between galectin-3 and TREM2, a solid-phase binding assay was conducted, which demonstrated a concentration-dependent binding of galectin-3 to TREM2-Fc (Fig. 3K). These findings suggest that galectin-3 is a ligand for TREM2 by interacting with the galectin-3-CRD and TREM2-Ig domains.

### Galectin-3 inhibits the TREM2/DAP12 receptor complex to suppress the Src-Syk signaling pathway and alter macrophages to an M2-like phenotype

**Fig. 4:**
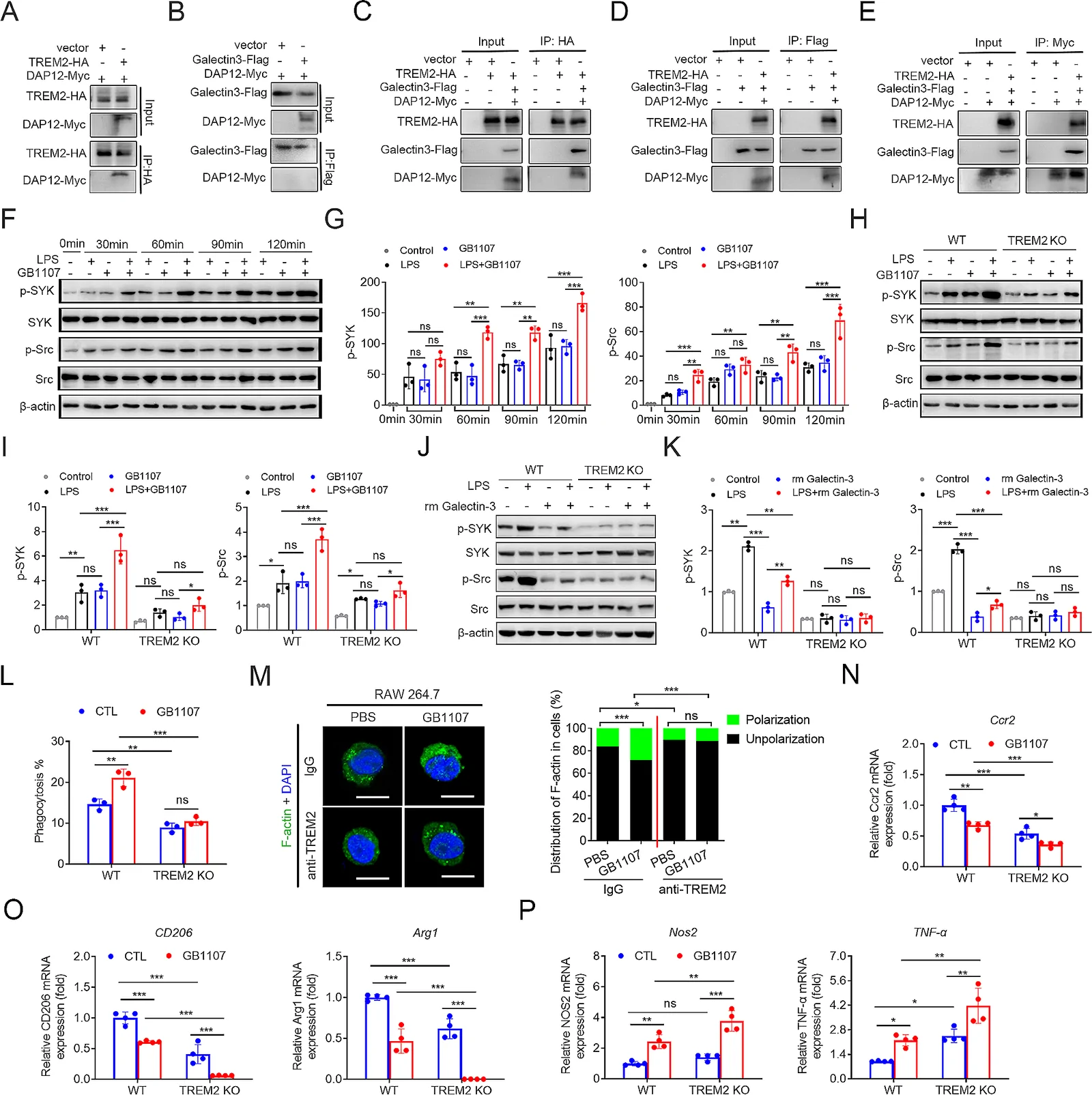
Galectin-3 inhibits TREM2/DAP12 receptor complex to suppress Src/Syk signaling pathway and altered macrophage to an M2-like phenotype. **(A**,** B**) 293T cells were transfected with plasmids as shown, and anti-HA (**B**) or anti-Flag (**B**) antibodies were employed for exogenous CO-IP experiments. (**C-E**) 293T cells were transfected with plasmids as shown and anti-HA (**C**), anti-Flag (**D**), or anti-Myc (**E**) antibodies were employed for exogenous CO-IP experiments, respectively. **(F**,** G**) After 24 h of LLC CM treatment with the addition of GB1107 (5 µM), and 2 h of stimulation with LPS (1 ng/mL), the phosphorylation levels of Src and Syk in indicated time point were analyzed by western blot (**F**). The gray values of p-Syk and p-Src protein bands were analyzed by Image J software, and the relative gray values were standardized to the gray values of Syk and Src (**G**). **(H**,** I**) After 24 h of LLC CM treatment with the addition of GB1107 (5 µM), and 2 h of stimulation with LPS (1 ng/mL), the phosphorylation levels of Src and Syk in WT or TREM2 KO BMDMs were analyzed by western blot (**H**). The gray values of p-Syk and p-Src protein bands were analyzed by Image J image analyses software, and the relative gray values were standardized to the gray values of Syk and Src (**I**). **(J**,** K**) After 24 h of LLC CM treatment with the addition of rm galectin-3 (200 ng/ml), and 2 h of stimulation with LPS (1 ng/mL), the phosphorylation levels of Src and Syk in WT or TREM2 KO BMDMs were analyzed by western blot (**J**). The gray values of p-Syk and p-Src protein bands were analyzed by Image J image analyses software, and the relative gray values were standardized to the gray values of Syk and Src (**K**). (**L**) Phagocytosis assay between WT or TREM2 KO BMDMs which were pretreated with LLC CM supplemented with GB1107 (5 µM) for 24 h and LLC were detected by flow cytometry. (**M**) Following a 24 h-treatment with LLC CM supplemented with GB1107 (5 µM), the F-actin polarization of RAW264.7 cells blocked with anti-TREM2 antibody was observed by confocal microscopy. Scale bars, 5 μm. Quantitative statistics of F-actin polarization were analyzed by Image J image analyses software. (**N-P**) After 24 h of LLC CM which was supplemented with GB1107 (5 µM) treatment, the transcription levels of *Ccr2*(**N**), M2-like macrophage markers (*CD206*, *Arg1*) (**O**) and M1-like macrophage markers (*Nos2*, *TNFα*) (**P**) in WT or TREM2 KO BMDMs were detected by RT-qPCR assay. Data represent mean ± SD from three experiments. *, *P* < 0.05; **, *P* < 0.01; ***, *P* < 0.001. ns, no significance

TREM2 signaling in myeloid cells is mainly dependent on the adaptor DAP12, which mediates intracellular signaling via the protein tyrosine kinase Syk [38]. Although the role of the TREM2-DAP12 receptor complex in lung cancer progression is unknown, our findings elucidated that TREM2 or DAP12 deficiency inhibited lung cancer progression in vivo (Supplementary Fig. 4A and 4B), suggesting that TREM2 affects lung cancer progression through the adaptor molecule, DAP12. Moreover, the relationship between galectin-3 and the TREM2-DAP12 signaling remains unclear. Exogenous CO-IP experiments confirmed the TREM2-DAP12 interaction (Fig. 4A); however, there was no interaction between galectin-3 and DAP12 (Fig. 4B). Subsequently, CO-IP experiments among TREM2-HA, galectin3-Flag, and DAP12-Myc were performed. The results revealed the detectability of any two proteins in precipitates enriched with anti-HA, anti-Flag, or anti-Myc antibodies (Fig. 4C and E), indicating that these three proteins can bind to form a complex that regulates downstream signaling pathways. TREM2 promotes phagocytosis in bacteria and apoptotic neurons through the DAP12-Src-Syk signaling pathway [38]. However, whether macrophages phagocytose tumor cells through the DAP12-Src-Syk signaling pathway is unknown. By examining the phosphorylation levels of Src and Syk in the galectin-3 inhibitor GB1107, or LPS (as a positive control) [39] treated-BMDMs which were pre-stimulated with LLC CM, we found that GB1107 activated the phosphorylation levels of Src and Syk over time in WT BMDMs (Fig. 4F and G). However, the activation of p-Src and p-Syk phosphorylation by GB1107 was significantly reduced in TREM2 KO BMDMs (Fig. 4H and I). Moreover, treatment with recombinant mouse galectin-3 protein (rm galectin-3) reduced the activation levels of p-Src and p-Syk phosphorylation in WT BMDMs, whereas no significant effect was observed in TREM2 KO BMDMs (Fig. 4J and K), suggesting that galectin-3 inhibited the Src-Syk signaling pathway downstream of TREM2.

Galectin-3 promotes tumor growth by inhibiting phagocytosis, macrophage re-polarization and T-cell immune responses [40]. However, whether galectin-3 affects TREM2-mediated phagocytosis and macrophage repolarization remains unclear. The findings from the phagocytosis assay indicated that the galectin-3 inhibitor GB1107 enhanced the phagocytosis of LLC CM-stimulated WT BMDMs, but had no significant effect on TREM2 KO BMDMs (Fig. 4L). Phagocytosis by macrophages ultimately leads to alterations in cytoskeletal proteins, known as actin polarization [41]. Further actin polarization experiments showed that GB1107 induced significant actin polarization in RAW264.7 cells, but blockade of TREM2 markedly attenuated actin polarization (Fig. 4M), suggesting that galectin-3 inhibition promotes TREM2-mediated actin polarization. Moreover, GB1107 treatment or TREM2 deficiency significantly inhibited the expression of *Ccr2*(Fig. 4N) and M2-like phenotypic markers (*CD206* and *Arg1*) (Fig. 4O), but markedly promoted M1-like phenotypic markers *(Nos2* and *TNFα*) expression (Fig. 4P) in both LLC CM-stimulated WT and TREM2 KO BMDMs. These results indicate that galectin-3 in tumor cell-CM inhibits TREM2-mediated phagocytosis and could synergizes with TREM2 to polarize macrophages to an M2-like phenotype.

### TREM2 promotes lung cancer progression via increasing M2-like macrophages and suppressing T/NK cell-mediated anti-tumor immune responses in vivo

**Fig. 5:**
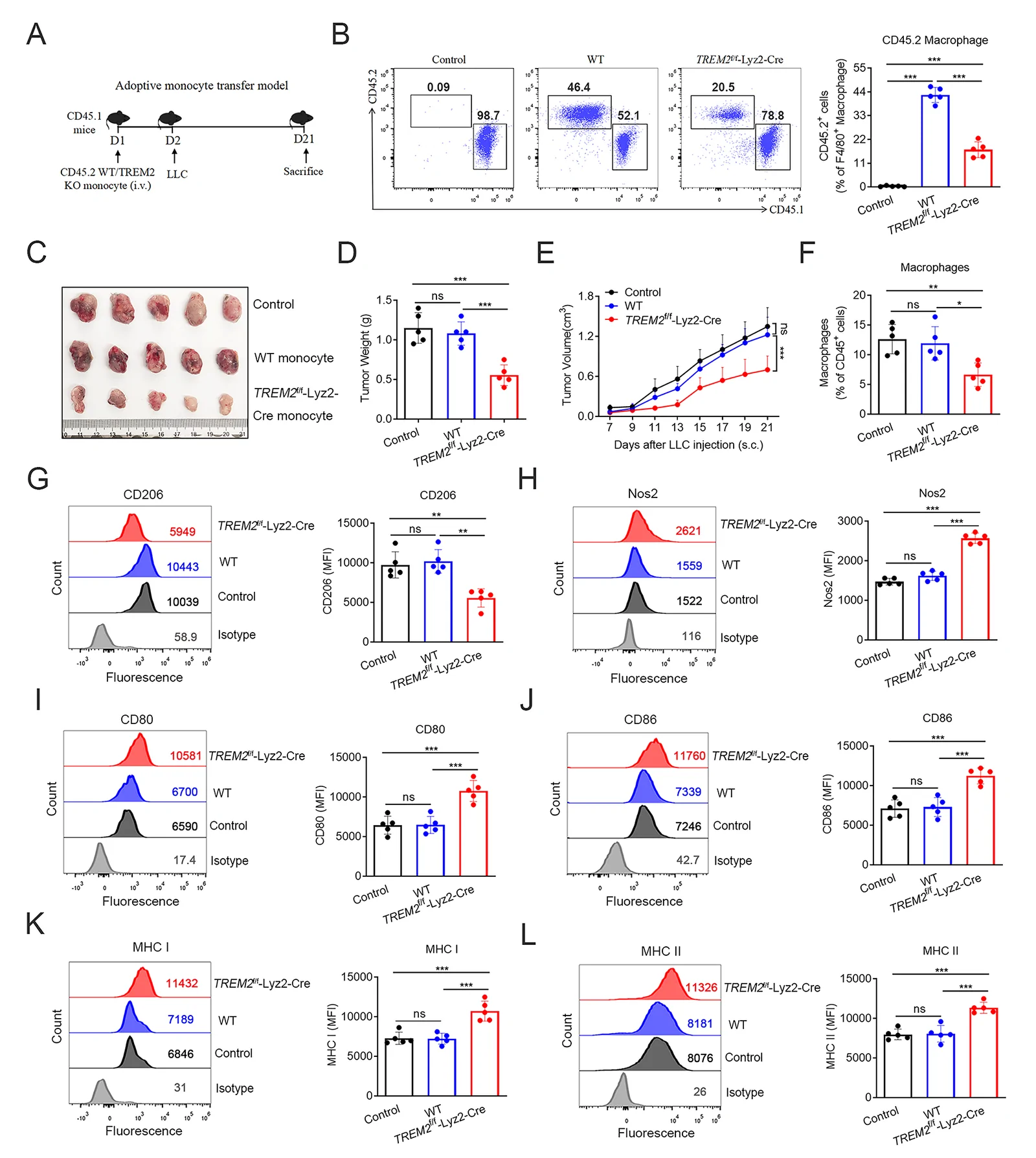
TREM2 promotes lung cancer progression and promotes M2-like immunosuppressive macrophage infiltration in the TME. (**A**) Diagram illustrating the establishment of a subcutaneous lung cancer model in CD45.1 mice injected with CD45.2 WT or TREM2 KO monocytes (*n* = 5). (**B**) Representative flow plots and quantification of CD45.2^+^ macrophages in tumor-infiltrating macrophages. (**C**) Tumor size of each group. (**D**) tumor weight of each group. (**E**) Tumor growth rate of each group. (**F**) Proportion of tumor-infiltrating macrophages was detected by flow cytometry. (**G-L**) Representative flow plots and quantification of CD206 (**G**), Nos2 (**H**), CD80 (**I**), CD86 (**J**), MHC I (**K**), and MHC II (**L**) in tumor-infiltrating macrophages of each group. The data represent the mean fluorescence intensity (MFI) of the specified molecule. Data represent mean ± SD from three experiments. *, *P* < 0.05; **, *P* < 0.01; ***, *P* < 0.001. ns, no significance

**Fig. 6:**
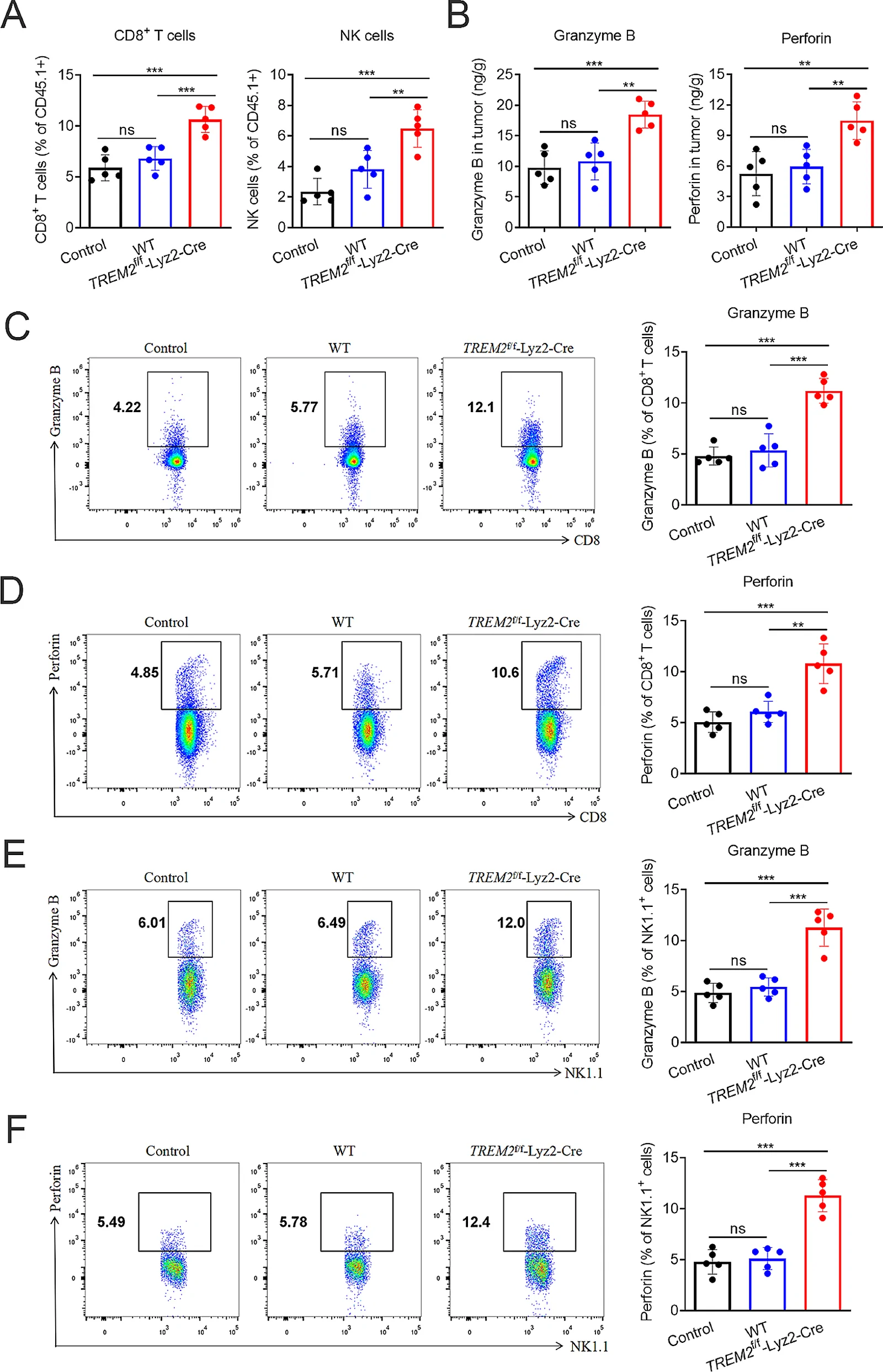
TREM2 impaired the infiltration and functionality of anti-tumor CD8^**+ **^T and NK cells in vivo. (**A**) In the adoptive monocyte transfer model, the proportion of anti-tumor CD8^+^ T and NK cells was detected by flow cytometry. (**B**) The concentrations of perforin and granzyme B in the tumor tissue grinding supernatant were detected by ELISA. (**C-D**) Representative flow plots and quantification of granzyme B (**C**) and perforin (**D**) producing CD8^+^ T cells in the TME. (**E-F**) Representative flow plots and quantification of granzyme B (**E**) and perforin (**F**) producing NK cells in the TME. Data represent mean ± SD from three experiments. **, *P* < 0.01; ***, *P* < 0.001. ns, no significance

To explore the regulatory function of TREM2 on mononuclear macrophages in tumor progression, we established a monocyte co-injection model in CD45.1 mice [30] (Fig. 5A). The proportion of tumor-infiltrating CD45.2^+^ macrophages was notably increased in the group injected with WT monocytes compared to the group injected with TREM2 KO monocytes (Fig. 5B), suggesting that TREM2 promotes the recruitment of circulating monocytes to the TME. In addition, TREM2 KO monocyte-injected mice had smaller tumor volumes and weights and slower tumor growth than those of WT monocyte-injected mice (Fig. 5C and E), suggesting that TREM2 loss-of-function myeloid cells inhibit lung cancer progression. In addition, tumor-infiltrating immune cells analyzed by flow cytometry indicated that the proportion of M2-like macrophages was reduced in mice receiving TREM2 KO monocytes (Fig. 5F), whereas the proportions of CD8^+^ T and NK cells were elevated (Fig. 6A). Furthermore, TREM2 induced the M2-like TAMs in vivo by increasing the expression of CD206 (Fig. 5G), while decreasing the expression of Nos2 (Fig. 5H). Moreover, TREM2 inhibited macrophage-expressed antigen-presenting molecules MHC-I/II (Fig. 5K and L) and the coactivators CD80/CD86 (Fig. 5I and J), suggesting that TREM2 also affects T cell-mediated anti-tumor immune response. Therefore, we then analyzed the function of tumor-infiltrating T cells and found that TREM2 inhibited T cell-mediated secretion of anti-tumor molecules, including granzyme B and perforin (Fig. 6C and D). Moreover, TREM2 inhibited NK cell-mediated secretion of anti-tumor molecules, including granzyme B and perforin (Fig. 6E and F), which was consistent with the previous findings that TREM2^+^ mononuclear macrophages suppressed NK cell accumulation and cytolytic activity in lung cancer [29]. Similarly, ELISA results showed consistently increased granzyme B and perforin in the tumor-grinding fluid of mice injected with TREM2 KO monocytes (Fig. 6B), indicating that TREM2 promotes tumor growth in vivo by increasing the number of M2-TAMs and inhibiting the anti-tumor function of CD8^+^ T and NK cells.

### Combination therapy with TREM2 knockout and the galectin-3 inhibitor GB1107 significantly inhibits lung cancer progression

**Fig. 7:**
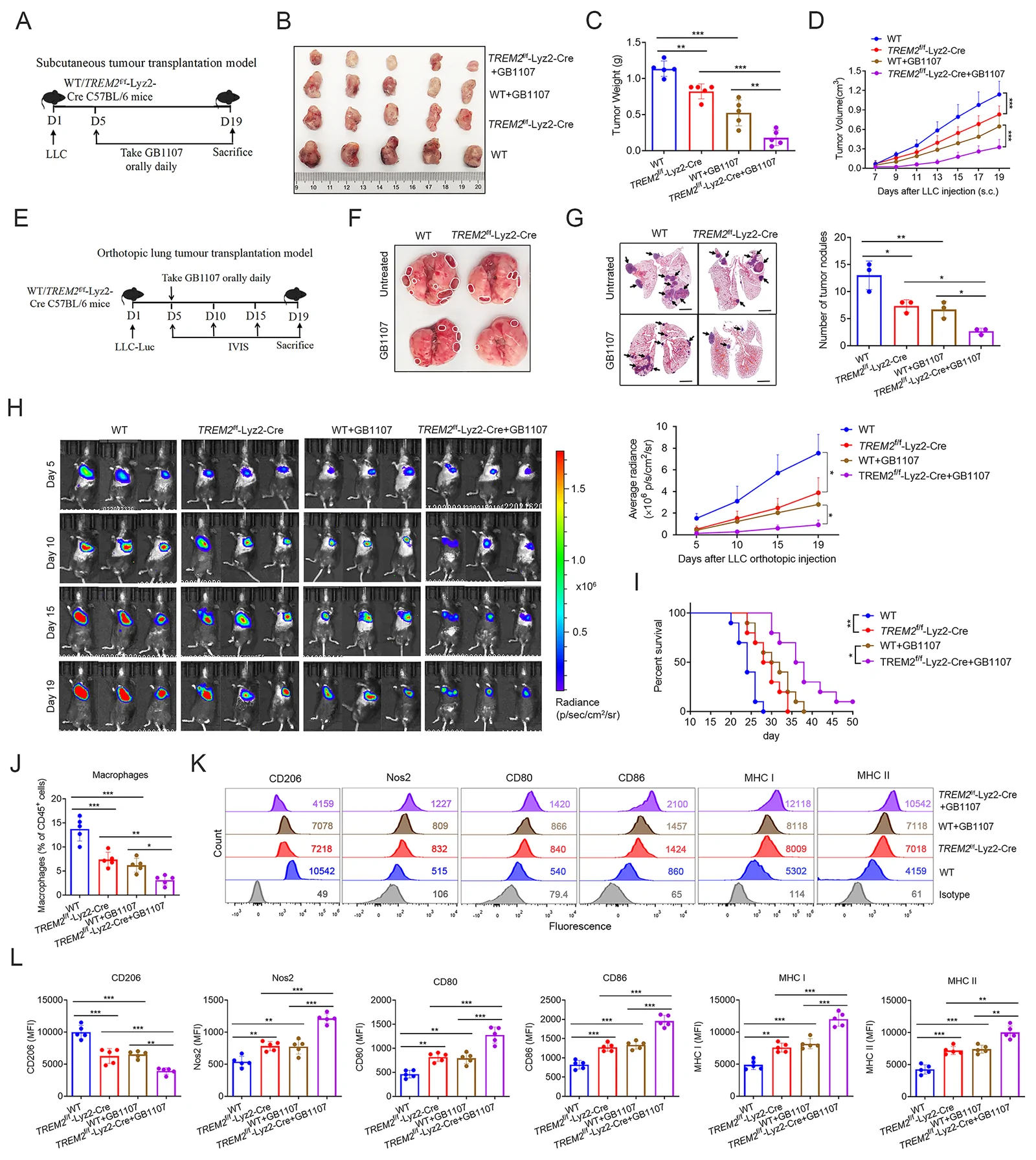
Combination therapy of the galectin-3 inhibitor GB1107 and TREM2 deficiency significantly inhibit lung cancer progression and reduced the immunosuppressive M2-like TAMs infiltration. (**A**) Schematic representation of an immunocompetent subcutaneous lung cancer model using the murine lung cancer cell line LLC (*n* = 5). Mice were orally administered GB1107 (10 mg/kg) daily beginning on day 5 until the mice were sacrificed on day 19. (**B**) Tumor size of each group. (**C**) Tumor weight of each group. (**D**) Tumor growth rate of each group. (**E**) Schematic illustration of an immunocompetent orthotopic lung cancer model using LLC-luc (*n* = 3). Mice were orally administered GB1107 (10 mg/kg) daily beginning on day 5 until the mice were sacrificed on day 19. (**F**) Representative images of tumorigenesis in each group. (**G**) Representative HE staining of lung tissues from each group and statistical analyses of tumor nodules. (**H**) In vivo IVIS images of orthotopic lung cancer in each group at corresponding time points and results of quantitative fluorescence analyses. (**I**) Survival rate of orthotopic lung cancer in each group (*n* = 8). (**J**) In the orthotopic lung cancer model, the percentage of tumor-infiltrating macrophages was analyzed using flow cytometry. (**K-L**) Representative flow plots (**K**) and quantification (**L**) of CD206, Nos2, CD80, CD86, MHC I and MHC II in tumor-infiltrated macrophages in each group. The data is displayed as MFI. Data represent mean ± SD from three experiments. *, *P* < 0.05; **, *P* < 0.01; ***, *P* < 0.001

To further explore whether galactin-3-TREM2 interaction affects the progression of lung cancer, we established a subcutaneous transplantation tumor model of lung cancer in WT and *TREM2*^f/f^-Lyz2-Cre mice and administered GB1107 treatment daily (Fig. 7A). The results showed that *TREM2*^f/f^-Lyz2-Cre mice exhibited reduced tumor volumes and weights, slower tumor growth, and the combination therapy with the galectin-3 inhibitor GB1107 significantly decreased the tumor burden (Fig. 7B and D). To further explore the effect of the galectin3-TREM2 interaction on lung cancer progression under physiological conditions, we constructed an orthotopic lung cancer model using luciferase-carrying LLC cells (LLC-luc) (Fig. 7E). The results demonstrated that TREM2 deficiency inhibited lung tumor formation and progression, which were further inhibited by GB1107 treatment (Fig. 7F and G). Furthermore, dynamic observations using IVIS fluorescence imaging showed that TREM2 deficiency inhibited lung tumor growth, which was markedly suppressed by GB1107 treatment (Fig. 7H). Moreover, survival analyses revealed that TREM2 deficiency prolonged the survival time of tumor-bearing mice, and combination treatment with GB1107 considerably improved their lifespan (Fig. 7I). These results indicated that TREM2 deficiency inhibits lung cancer progression, which can be further inhibited by galectin-3 inhibition.

**Fig. 8:**
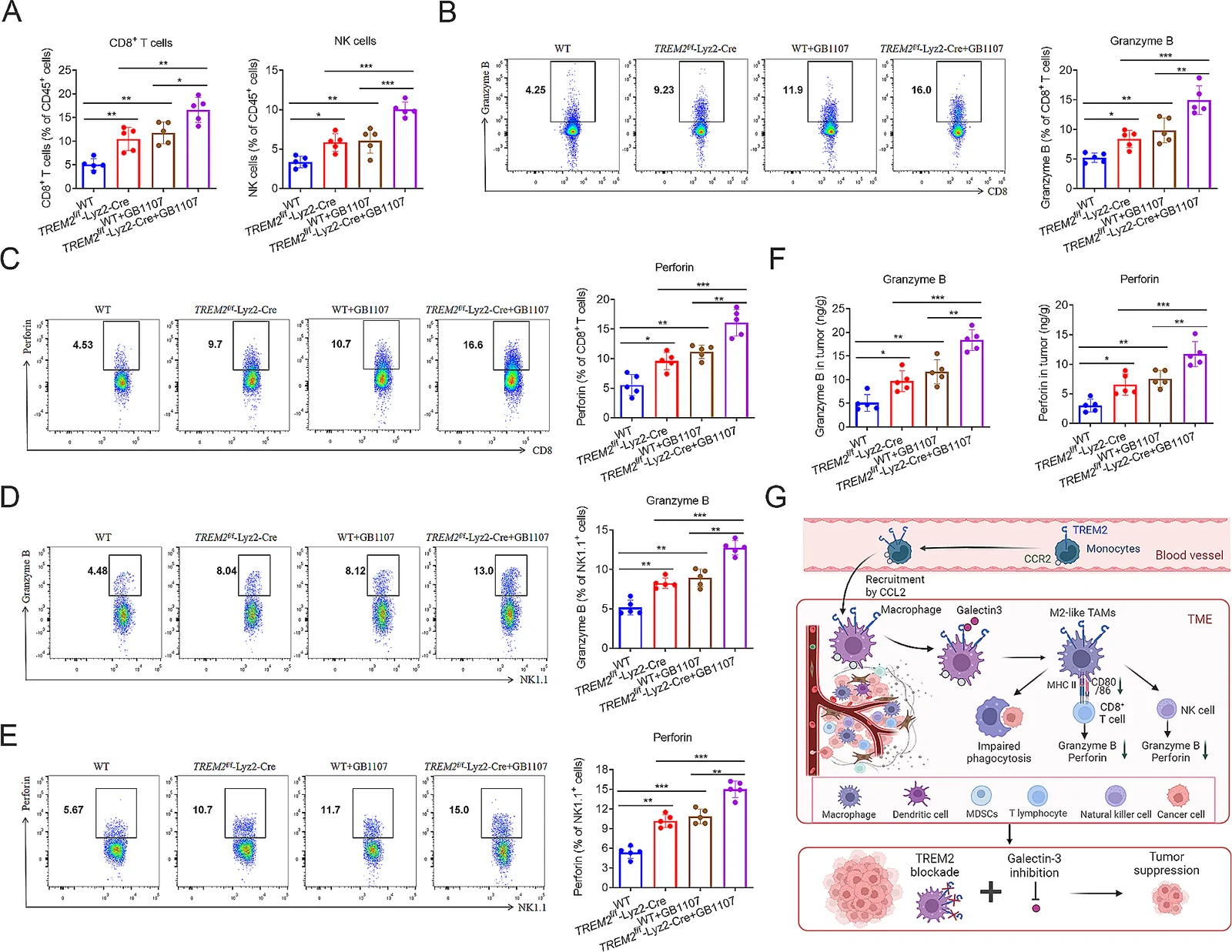
Combination of the Galectin-3 inhibitor and TREM2 deficiency enhanced the infiltration and functionality of anti-tumor CD8^**+ **^T and NK cells. (**A**) In the orthotopic lung cancer models, the percentage of anti-tumor CD8^+^ T and NK cells were analyzed using flow cytometry. (**B-C**) Representative flow plots and quantification of granzyme B (**B**) and perforin (**C**) producing CD8^+^ T cells in TME. (**D-E**) Representative flow plots and quantification of granzyme B (**D**) and perforin (**E**) producing NK cells in TME. (**F**) The concentrations of perforin and granzyme B in the tumor tissue grinding supernatant were detected by ELISA. (**G**) A propose model to illustrate the mechanism of galetin3-TREM2 axis in promoting lung cancer progression. Data represent mean ± SD from three experiments. *, *P* < 0.05; **, *P* < 0.01; ***, *P* < 0.001

Flow cytometric analyses of tumor-infiltrating immune cells showed that the proportion of macrophages was decreased in *TREM2*^f/f^-Lyz2-Cre mice; however, the percentages of CD8^+^ T and NK cells were increased compared to those in WT mice, and were further decreased or increased in combination with GB1107 treatment, respectively (Figs. 7J and 8A). In addition, dual inhibition of TREM2 and galectin3 significantly increased the M2-like TAMs in vivo by promoting CD206 expression while inhibiting Nos2 expression (Fig. 7K and L). Furthermore, the dual blockade of TREM2 and galectin3 notably increased the expression of the coactivators CD80/86 (Fig. 7K and L) and antigen-presenting molecules MHC I/II (Fig. 7K and L) on TAMs. Moreover, tumor-infiltrating CD8^+^ T cells and NK cells in TREM2-deficient mice were more capable of secreting perforin and granzyme B, and GB1107 treatment further enhanced it (Fig. 8B and E). Similarly, ELISA results showed increased granzyme B and perforin in the tumor-grinding fluid of TREM2-deficient mice, which were further enhanced by GB1107 treatment (Fig. 8F). Collectively, these results suggest that dual blockade of galectin3 and TREM2 inhibit lung tumor growth in vivo by decreasing immunosuppressive macrophages and facilitating the anti-tumor function of CD8^+^ T and NK cells.

## Discussion

High infiltration of TAMs is a characteristic feature of the TME, not only in lung cancer but also in other cancers [42]. Peripheral circulating monocytes are usually recruited to the TME through various chemokine networks, such as CCL2, CCL7, and CCL13 [11]. Patrolling monocytes can be protective in the early stages of cancer. However, when bone marrow-derived monocytes gradually become the predominant subtype of TAMs, they often lead to the progression of primary and metastatic tumors [11]. Hendrikx T et al. found that bone marrow-derived monocytes recruited during steatohepatitis acquired high TREM2 expression in the circulation [43], suggesting that TREM2 may affect the circulating monocytes recruitment to the disease focus. In addition, increased TREM2 expression in TAMs promotes tumor progression in colorectal cancer [44], ovarian cancer [23], and TACE [24]. Our findings revealed a high TREM2 expression in lung cancer macrophages, and abundant TREM2^+^ macrophages are enriched in the intratumor site in the presence of the CCL2-CCR2 chemotactic axis. Importantly, TREM2 deficiency inhibits lung cancer progression in vivo by reducing the population of M2-like TAMs and enhancing both the quantity and functionality of CD8^+^ T and NK cells. As far as we know, this study represents the first investigation to document that TREM2 affects TAMs infiltration by regulating the chemotactic axis, paving the way for new therapeutic avenues to inhibit cancer development.

TAMs are highly plastic and heterogeneous cell populations in the TME that make up to 50% of certain solid tumors [45]. The significance of TAMs in tumor immunity has garnered notable interest in recent years. TAMs acquire a polarized M2-like phenotype, which involves in subverting adaptive immunity and promoting tumor growth and progression. However, TAMs can mediate anti-tumor effects (M1-like phenotype) through immunosurveillance, including phagocytosis and innate immune sensing. Current TAMs-targeting therapies include (1) alteration of the constitution of TAMs, (2) immunosuppressive TAMs blockade, (3) reprogramming of M2-TAMs, and (4) novel targets, such as TREM2, Siglec-15, and MARCO [46]. Among them, notable advancements have been made in reprogramming pro-tumor M2-like TAMs, which have impaired phagocytic activity, into anti-tumor M1-like TAMs with robust phagocytic activity [47]. As a surface receptor, TREM2 plays a significant part in macrophage-mediated tumor immune responses, including phenotypic switching and phagocytosis [48]. TREM2 promotes phagocytosis of a series of damaged substances in the organism and foreign objects in a non-specific manner [16]. In addition, the absence of TREM2 hampers the capacity of myeloid cells within glioma to engulf tumor cells [27]. Our findings revealed that TREM2 promotes macrophage-mediated phagocytosis of tumor cells but is inhibited upon treatment with LLC CM. Furthermore, LLC CM management promotes the conversion of TREM2^+^ macrophages to a tumor-promoting M2-like phenotype, suggesting that special molecules in tumor cell-derived CM could regulate the physiological function of TREM2, such as impairing TREM2-mediated phagocytosis.

TREM2 exerts multiple functions by binding to a range of ligands, primarily anionic molecules, including bacterial products, lipids (APOE, and CLU/APOJ), and anionic molecules [49], but the identification of highly specific TREM2 ligands is largely unknown. Krasemann S et al. reported that the TREM2-APOE pathway serves as the primary regulator of phenotypic alterations in microglia during neurodegenerative diseases, making it a potential target for reinstating microglial homeostasis [49]. Moreover, the co-localization of TREM2 and galectin-3 has been identified in AD-associated microglia [18], suggesting that galectin-3 may be a potential endogenous TREM2 ligand which requires further validation. The results of liquid chromatography-mass spectrometry revealed that soluble galectin3 is a potential ligand of TREM2 in TAMs. Exogenous/endogenous CO-IP experiments and immunofluorescence experiments demonstrated the interaction and co-localization of TREM2 and galectin-3. A solid phase binding assay further confirmed their direct binding. TREM2 consists of a short extracellular structural domain, a transmembrane helix, and a short cytoplasmic tail that lacks signal transduction or transportation motifs [16]. Galectin-3 consists of a short NH2-terminal structural domain, a repetitive collagen alpha-like sequence, and a single CRD consisting of 140 amino acids. Furthermore, CO-IP results demonstrated that the TREM2-galectin-3 interaction was dependent on the TREM2-Ig and galectin-3-CRD domains. These data elucidate that galectin-3 is a ligand for TREM2 in TAMs.

Galectin-3 is a multifunctional protein of the beta-galactosidase-binding protein family [19]. Galectin-3 plays crucial roles in cancer, including promotion of tumor cell survival, angiogenesis, tumor transformation, tumor progression and metastasis [50]. The unique structure of galectin-3 allows it to interact with an excess of ligands, such as laminin and fibronectin in a carbohydrate-dependent or independent manner [51]. Galectin-3 has a high expression level in NSCLC patients [52]. Galectin-3 can promote macrophage differentiation to an M2-like phenotype and inhibit CD8^+^ T cell-mediated anti-tumor effects; therefore, galectin-3 deficiency or pharmacological blockade can significantly inhibit lung cancer progression [20]. Similarly, we found that galectin-3 expression was notably elevated in both the TME of lung adenocarcinoma patients and in the supernatant of LLC. Functionally, the galectin-3 inhibitor GB1107 alleviated the inhibitory effect of tumor cell-CM on TREM2-mediated phagocytosis. Moreover, the galectin-3 inhibitor GB1107 reversed the LLC CM-induced and TREM2-dependent M2-like macrophages transformation, suggesting that the galectin-3-TREM2 interaction may affect the downstream signaling pathway of TREM2, thereby affecting the function and phenotype of macrophages.

TREM2 signal transduction in macrophages primarily depends on the cytoplasmic adapter protein DAP12. DAP12 mediates the functions of TREM2, such as phagocytosis, by recruiting and phosphorylating downstream Src and Syk tyrosine kinases [16]. Herein, we found that TREM2-mediated phagocytosis of tumor cells was dependent on the DAP12-Src-Syk signaling pathway, which was inhibited by galectin-3 in LLC CM. Therefore, the galectin-3-TREM2 interaction inhibits the phosphorylation of Src and Syk downstream of TREM2 signaling and is involved in suppressing phagocytosis and promoting the M2-like phenotype switching of macrophages. The exact downstream signaling pathways and mechanisms by which TREM2 downstream molecules Src and Syk induce macrophage phenotypic transformation and phagocytosis remain unclear. Notably, combination therapy with TREM2 knockout and the galectin-3 inhibitor GB1107 substantially inhibited lung cancer progression in vivo by decreasing tumor-infiltrating M2-like macrophages and increasing antitumor CD8^+^ T and NK cells. Moreover, combination therapy upregulates antigen-presenting factors, such as MHC I/II and the coactivators CD80/86 in macrophages, promoting adaptive anti-tumor immune responses.

In summary, our findings elucidate that elevated TREM2 expression is correlated with increased infiltration of immunosuppressive M2-like TAMs and suppression of intratumoral CD8^+^ T and NK cell responses in lung cancer. TREM2^+^ TAMs are recruited to the intratumor area through the CCL2-CCR2 chemotactic axis from peripheral TREM2^+^ monocytes. Tumor-educated TREM2^+^ macrophages are converted into M2-like TAMs by interacting with the novel ligand galectin-3 enriched in the TME. Notably, TREM2 knockout combined with the galectin-3 inhibitor GB1107 substantially inhibited the immunosuppressive effect of M2-TAMs and enhanced T/NK cell-mediated anti-tumor effects, improving the efficacy of lung cancer therapy (Fig. 8G). Overall, we provide molecular and cellular insights into the manipulation of tumor progression by TREM2^+^ TAMs and propose a valuable approach for remodeling the anti-tumor TME, emphasizing that TREM2 blockade in combination with galectin-3 inhibitors may present a practical strategy for the clinical management of lung cancer or other cancer types.

## Electronic supplementary material

Below is the link to the electronic supplementary material.

Supplementary Material 1: Supplementary Information includes supplementary figures (Supplementary Figs. 1–4) and supplementary tables (Supplementary Tables 1–3).

## Data Availability

Data confirming the results of this study are presented in the manuscript and are available from the corresponding author upon reasonable request. The single-cell RNA sequencing data included in this study were obtained from the Gene Expression Omnibus (GEO) (NIH, RRID: SCR_005012) database of the National Centre for Biotechnology Information (NCBI), with the accession number GSE123904. Source data are provided with this paper.

## Abbreviations

TAMs: Tumor-associated macrophages
TREM2: Triggering receptor expressed on myeloid cells 2
TME: Tumor microenvironment
NSCLC: Non-small cell lung cancer
DAP12: DNAX activation protein 12
CM: Conditioned medium
GEO: Gene Expression Omnibus
