# Correction: Galectin-3 induces pathogenic immunosuppressive macrophages through interaction with TREM2 in lung cancer

**DOI:** 10.1186/s13046-026-03831-2

**Published:** 2026-10-01

**Authors:** Qiaohua Wang, Yongjian Wu, Guanmin Jiang, Xi Huang

**Affiliations:** 1 Center for Infection and Immunity, Guangdong Provincial Engineering Research Center of Molecular Imaging, the Fifth Affiliated Hospital, Sun Yat-sen University, Zhuhai, China; 2 Department of Clinical Laboratory, the Fifth Affiliated Hospital, Sun Yatsen University, Zhuhai, China; 3 Guangdong-Hong Kong-Macao University Joint Laboratory of Interventional Medicine, the Fifth Affiliated Hospital, Sun Yat-sen University, Zhuhai, China; 4 Zhuhai Engineering Research Center of Infection and Immunity, the Fifth Affiliated Hospital, Sun Yat-sen University, Zhuhai, China

**Correction: J Exp Clin Cancer Res 43, 224 (2024)**

**https://doi.org/10.1186/s13046-024-03124-6**

Following publication of the original article [1], the authors identified errors in Fig. 5B and 7H, where an image from a different experimental batch or group was inadvertently used.

The incorrect and the correct figures are given below.

The incorrect figure 5 was

**Figure:**
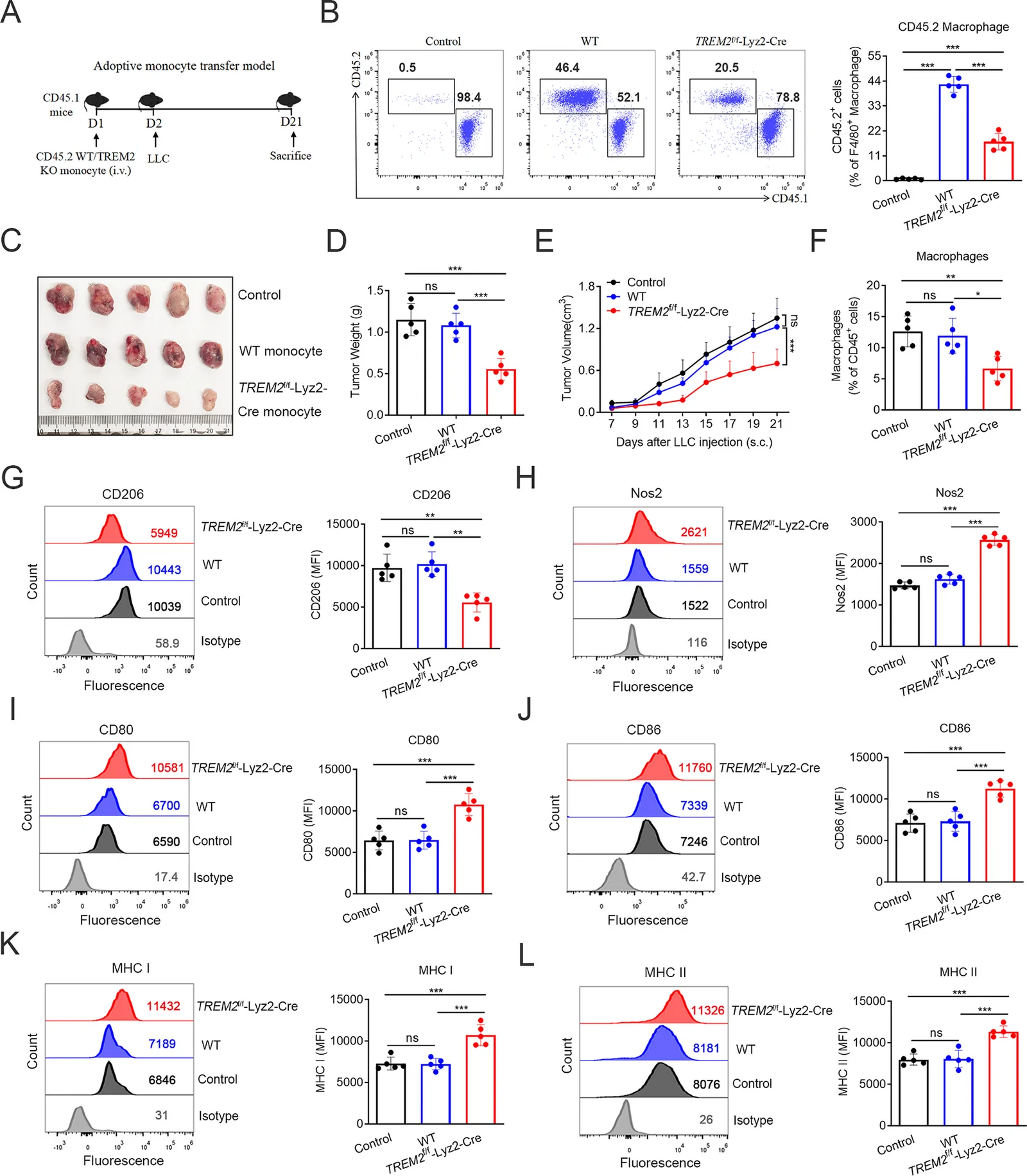

The correct figure 5 is

**Figure:**
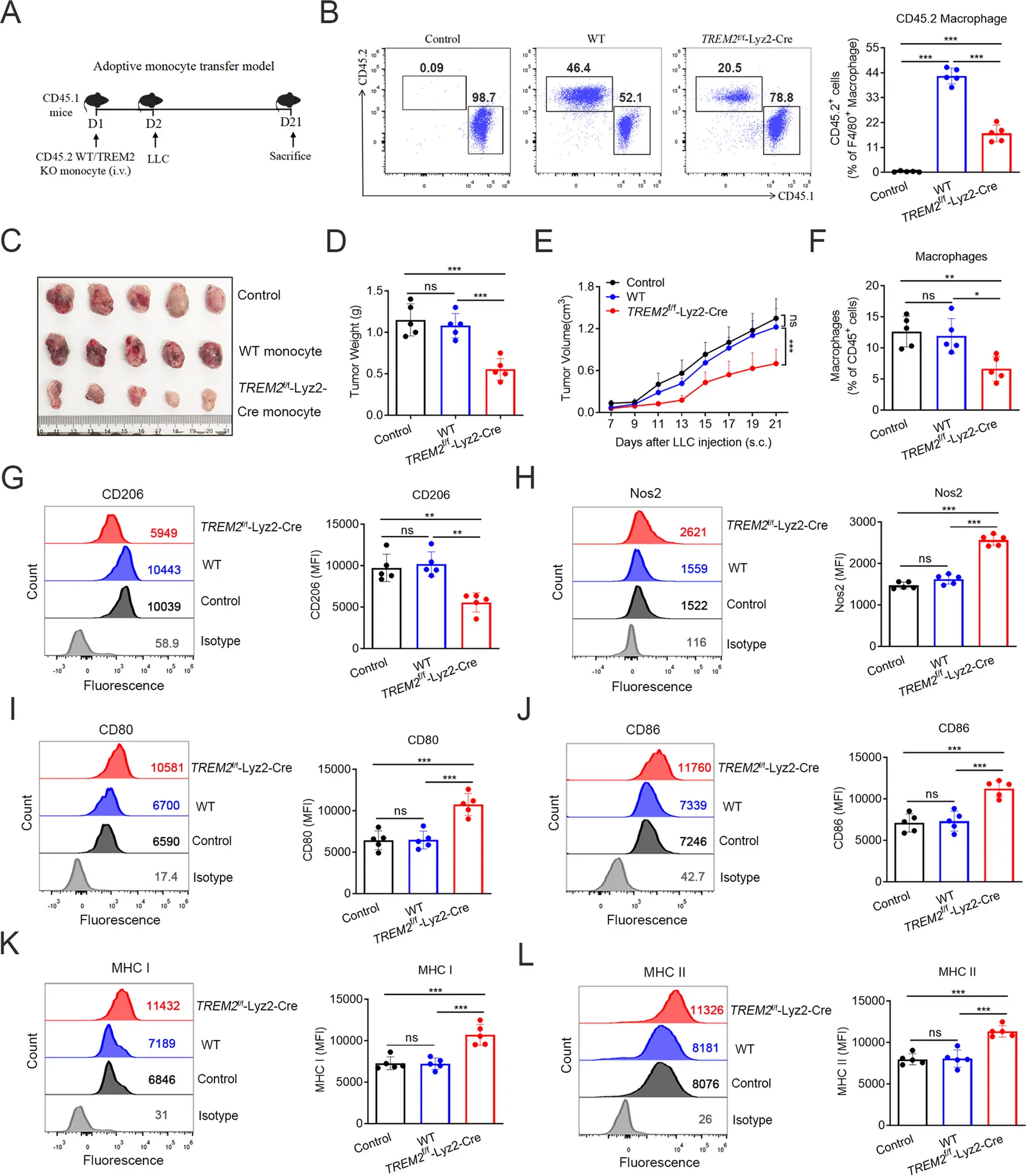

The incorrect figure 7 was

**Figure:**
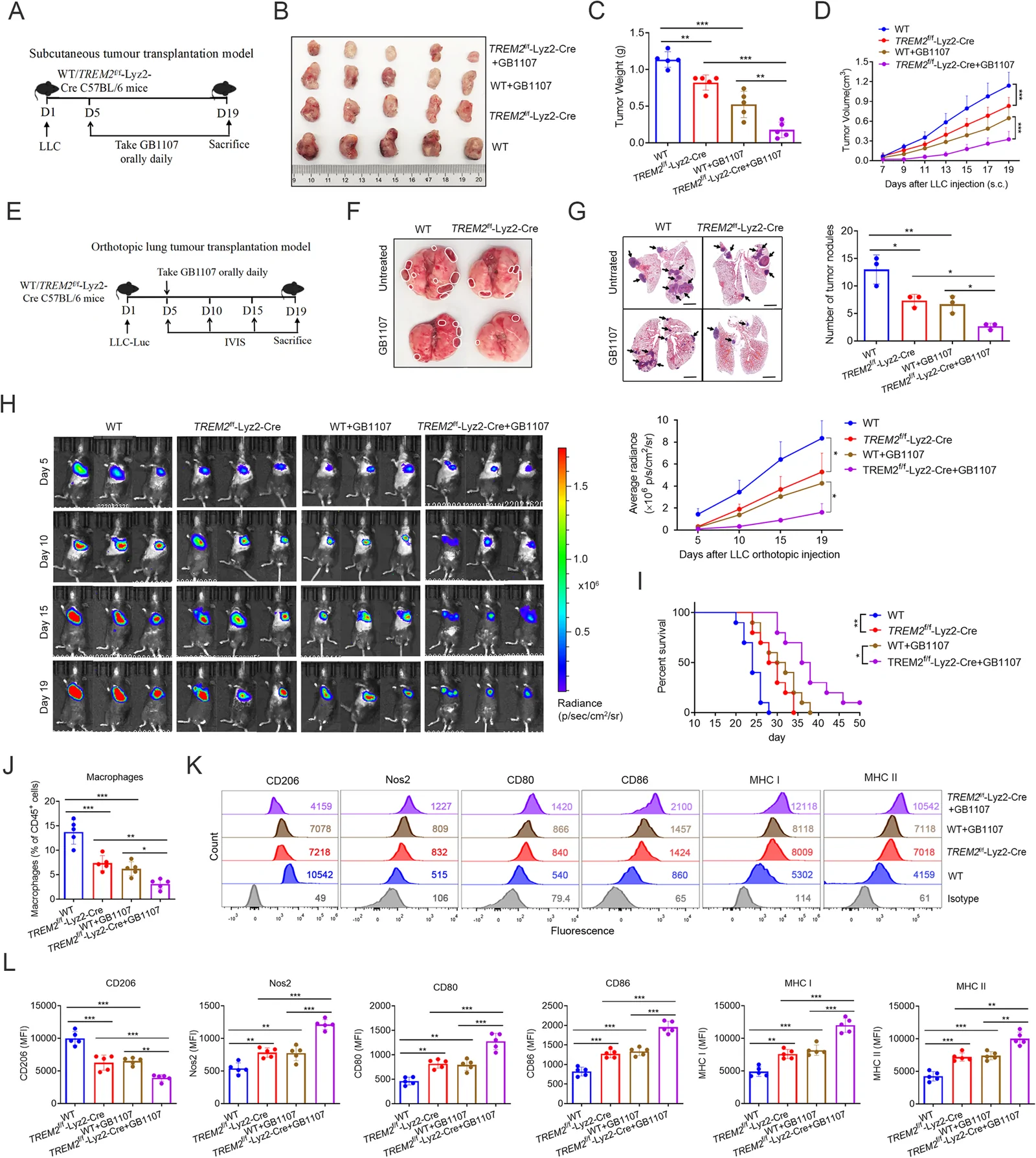

The correct figure 7 is

**Figure:**
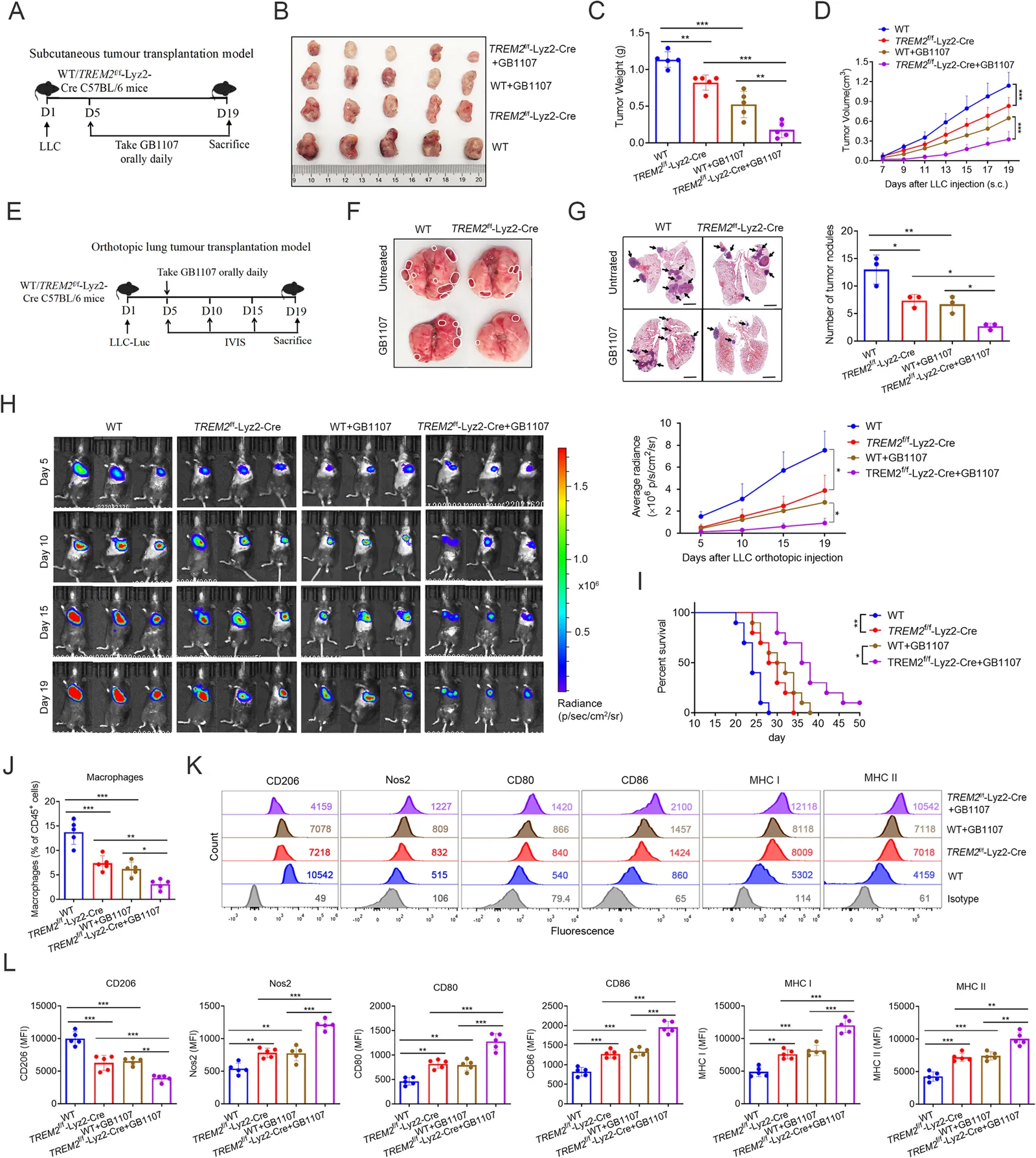

This correction does not affect the scientific integrity or conclusions of the study.

The original article has been updated.
