# Supplementary material for: Galectin-3 induces pathogenic immunosuppressive macrophages through interaction with TREM2 in lung cancer
Source: J Exp Clin Cancer Res. 2024 Aug 13;43:224. doi: 10.1186/s13046-024-03124-6 (PMC11321020; doi:10.1186/s13046-024-03124-6)
Supplement: Supplementary file 1 — Supplementary Material 1: Supplementary Information includes supplementary figures (Supplementary Figs. 1–4) and supplementary tables (Supplementary Tables 1–3). [file 13046_2024_3124_MOESM1_ESM.docx]

Supplementary Material

1. **Supplementary Figures**

**
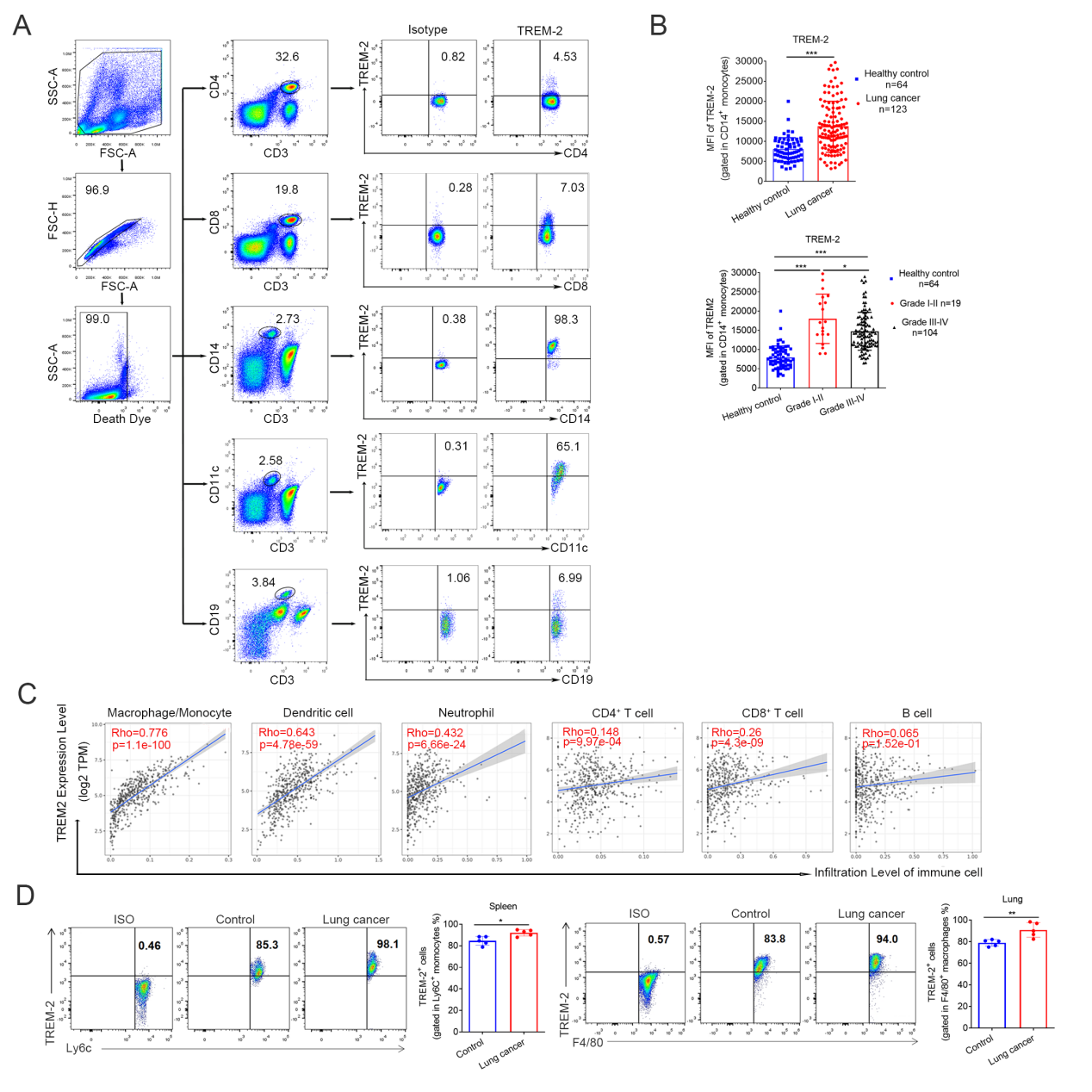
**

**Supplementary Figure 1. TREM2 expression is up-regulated in mononuclear macrophages of lung cancer.** **(A)** Flow cytometry loop gate strategy and expression levels of TREM2 in human peripheral blood immune cells of lung cancer patients and healthy donors. **(B)** The mean fluorescence intensity (MFI) of TREM2 expression on CD14^+^ monocytes from healthy donors (n=64) and lung cancer patients (n=123) with different TNM stages were analyzed by flow cytometry. **(C)** Correlation analyses between TREM2 expression abundance and tumor-infiltrating immune cells in lung cancer in the TCGA database. **(D)** The expression proportion of TREM2 in lung-infiltrating macrophages and spleen monocytes of lung cancer-bearing mice and healthy mice were detected by flow cytometry. Data represent mean ± SD from three experiments. *, *P* < 0.05; **, *P* < 0.01; ***, *P* < 0.001.

**
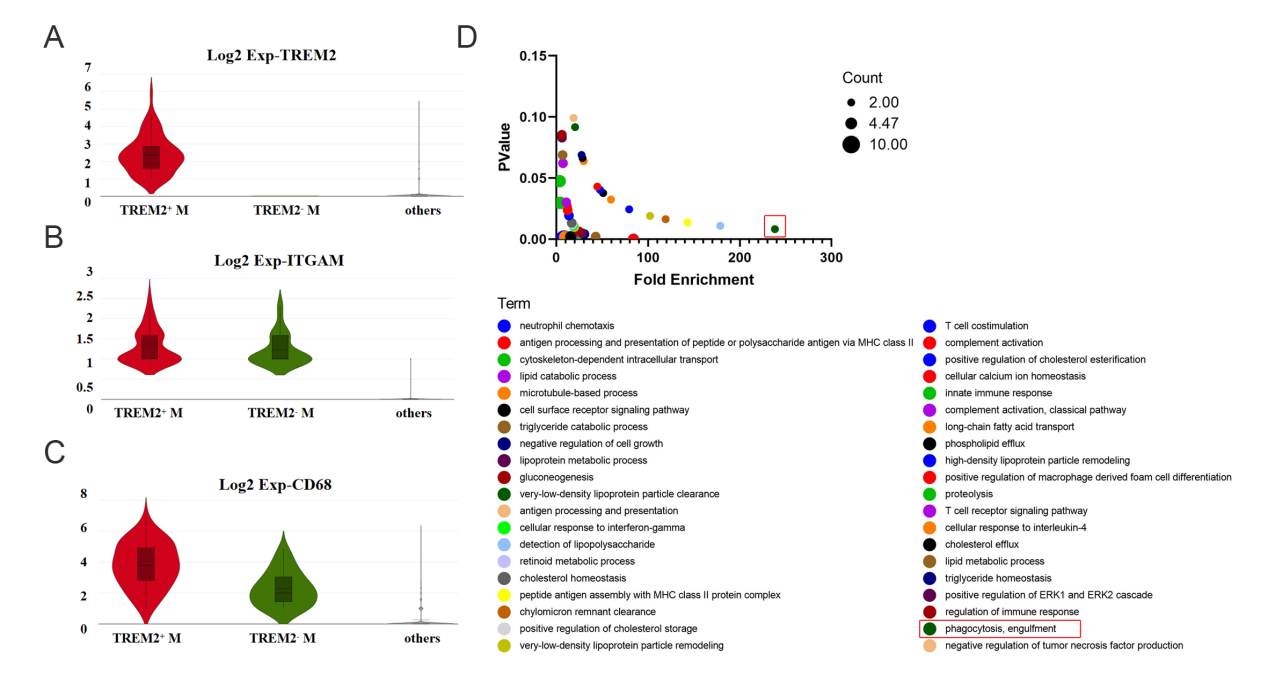
**

**Supplementary Figure 2. TREM2 in lung cancer-associated macrophages has a tendency to promote phagocytosis of tumor cells. (A-C)** Expression of TREM2 (A), ITGAM (CD11b) (B) and CD68 (C) in TREM2-positive and TREM2-negative macrophages in lung cancer patients. **(D)** GO-BP analyses of TREM2-positive and TREM2-negative macrophages shows the biological process enriched in TREM2-positive macrophages compared to TREM2-negative macrophages.

**
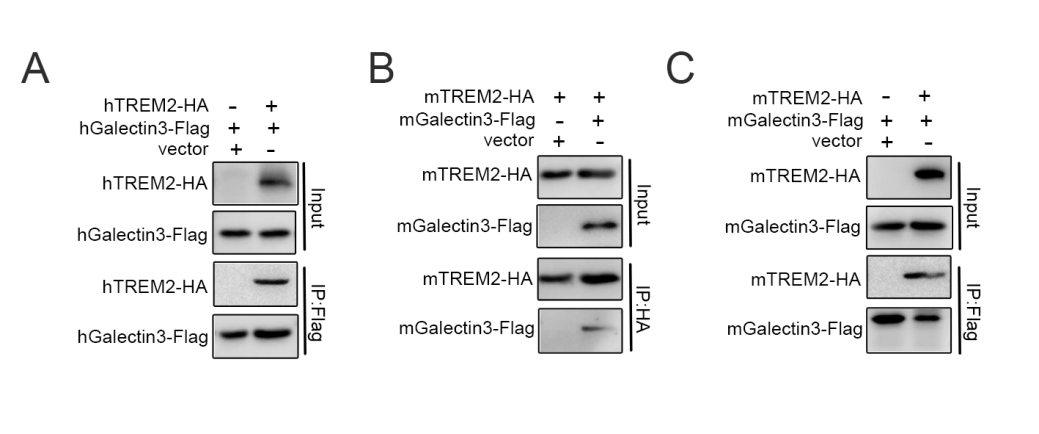
**

**Supplementary Figure 3. Tumor derived galectin-3 was a ligand for TREM2. (A)** 293T cells were transfected with pcDNA3.1-vector/pcDNA3.1-hTREM2-HA/pcDNA3.1-hGalectin-3-Flag plasmids as indicated. Exogenous CO-IP experiments were performed with anti-Flag antibody. **(B, C)** 293T cells were transfected with pcDNA3.1-vector/pcDNA3.1-mTREM2-HA/pcDNA3.1-mGalectin-3-Flag plasmids as indicated. Exogenous CO-IP experiments were performed with anti-HA **(B)** or anti-Flag **(C)** antibody.

**
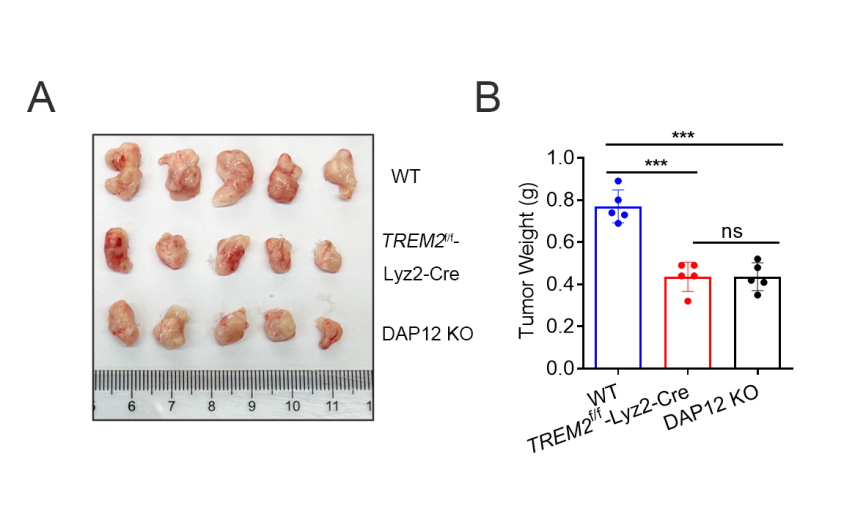
**

**Supplementary Figure 4. TREM2/DAP12 complex inhibited lung cancer progression *in vivo*.** **(A-B)** Subcutaneous transplantation tumor model of lung adenocarcinoma was established in WT, *TREM2*^f/f^-Lyz2-Cre and DAP12 KO mice (n=5). Tumor size of each group was exhibited in **(A)**. Tumor weight of each group was shown in **(B)**. Data represent mean ± SD from three experiments. ***, *P* < 0.001.

1. **Supplementary Tables**

**Supplementary Table 1.** Summary of clinical characteristics of lung cancer patients and healthy donors.

| Table S1: Summary of clinical characteristics of lung cancer patients and healthy donors. | | |
| --- | --- | --- |
| Characteristics | Lung cancer patients (n=123) | Healthy donors (n=64) |
| Age (years) | | |
| Medium | 60 | 54 |
| Range | 31-84 | 22-82 |
| <60 (% of all) | 44.5 | 46.9 |
| ≥60 (% of all) | 55.5 | 53.1 |
| Sex (% of all) | | |
| Male | 63.4 | 62.5 |
| Female | 36.6 | 37.5 |
| TNM stage (% of patients) | | |
| I | 4.1 | - |
| II | 11.4 | - |
| III | 21.9 | - |
| IV | 62.6 | - |
| Pathologic subtype (% of patients) | | |
| Adenocarcinoma | 69.9 | - |
| squamous cell carcinoma | 13.0 | - |
| Other | 17.1 | - |

**Supplementary** **Table 2.** RT-qPCR Primer sequences used in this study.

| Table S2. RT-qPCR Primer sequences used in this study. | | |
| --- | --- | --- |
| Gene name | Forward primer (5’-3’) | Reverse primer (5’-3’) |
| *Actin* | GATTACTGCTCTGGCTCCTAGC | GACTCATCGTACTCCTGCTTGC |
| *M-Nos2* | CACCACCCTCCTCGTTC | CAATCCACAACTCGCTCC |
| *M-CD206* | ATGGATTGCCCTGAACAGCA | TGTACCGCACCCTCCATCTA |
| *M-Arg1* | AAGACAGCAGAGGAGGTG | AGTCAGTCCCTGGCTTAT |
| *M-Tnfα* | GGTGCCTATGTCTCAGCCTCTT | GCCATAGAACTGATGAGAGGGAG |
| *M-Ccl2* | GGGTCCAGACATACATTAA | ACGGGTCAACTTCACATT |
| *M-Ccl3* | ACTGCCTGCTGCTTCTCCTACA | ATGACACCTGGCTGGGAGCAAA |
| *M-Ccl13* | GATCTCCTTGCAGAGGCTGAAG | TCTGGACCCACTTCTCCTTTGG |
| *M-Ccl17* | CGAGAGTGCTGCCTGGATTACT | GGTCTGCACAGATGAGCTTGCC |
| *M-Ccr2* | GCTGTGTTTGCCTCTCTACCAG | CAAGTAGAGGCAGGATCAGGCT |

**Supplementary** **Table 3.** Mass spectrometry identified proteins interacting with TREM2 in lung cancer macrophages.

| Table S3. Proteins identified by LC-MS mass spectrometry. (Top 20) | | |
| --- | --- | --- |
| Number | Accession | Description |
| 1 | P35579\|MYH9_HUMAN | Myosin-9 OS=Homo sapiens OX=9606 GN=MYH9 PE=1 SV=4 |
| 2 | P01834\|IGKC_HUMAN | Immunoglobulin kappa constant OS=Homo sapiens OX=9606 GN=IGKC PE=1 SV=2 |
| 3 | P08670\|VIME_HUMAN | Vimentin OS=Homo sapiens OX=9606 GN=VIM PE=1 SV=4 |
| 4 | P02675\|FIBB_HUMAN | Fibrinogen beta chain OS=Homo sapiens OX=9606 GN=FGB PE=1 SV=2 |
| 5 | P17931\|LEG3_HUMAN | Galectin-3 OS=Homo sapiens OX=9606 GN=LGALS3 PE=1 SV=3 |
| 6 | P08311\|CATG_HUMAN | Cathepsin G OS=Homo sapiens OX=9606 GN=CTSG PE=1 SV=2 |
| 7 | P04792\|HSPB1_HUMAN | Heat shock protein beta-1 OS=Homo sapiens OX=9606 GN=HSPB1 PE=1 SV=2 |
| 8 | P09429\|HMGB1_HUMAN | High mobility group protein B1 OS=Homo sapiens OX=9606 GN=HMGB1 PE=1 SV=3 |
| 9 | P01024\|CO3_HUMAN | Complement C3 OS=Homo sapiens OX=9606 GN=C3 PE=1 SV=2 |
| 10 | P04004\|VTNC_HUMAN | Vitronectin OS=Homo sapiens OX=9606 GN=VTN PE=1 SV=1 |
| 11 | P02792\|FRIL_HUMAN | Ferritin light chain OS=Homo sapiens OX=9606 GN=FTL PE=1 SV=2 |
| 12 | P07339\|CATD_HUMAN | Cathepsin D OS=Homo sapiens OX=9606 GN=CTSD PE=1 SV=1 |
| 13 | P02743\|SAMP_HUMAN | Serum amyloid P-component OS=Homo sapiens OX=9606 GN=APCS PE=1 SV=2 |
| 14 | O14950\|ML12B_HUMAN | Myosin regulatory light chain 12B OS=Homo sapiens OX=9606 GN=MYL12B PE=1 SV=2 |
| 15 | P0DMV8-2\|HS71A_HUMAN | Isoform 2 of Heat shock 70 kDa protein 1A OS=Homo sapiens OX=9606 GN=HSPA1A PE=1 SV=2 |
| 16 | P0C0L4\|CO4A_HUMAN | Complement C4-A OS=Homo sapiens OX=9606 GN=C4A PE=1 SV=2 |
| 17 | P08246\|ELNE_HUMAN | Neutrophil elastase OS=Homo sapiens OX=9606 GN=ELANE PE=1 SV=1 |
| 18 | P61626\|LYSC_HUMAN | Lysozyme C OS=Homo sapiens OX=9606 GN=LYZ PE=1 SV=1 |
| 19 | P02747\|C1QC_HUMAN | Complement C1q subcomponent subunit C OS=Homo sapiens OX=9606 GN=C1QC PE=1 SV=3 |
| 20 | Q05315\|LEG10_HUMAN | Galectin-10 OS=Homo sapiens OX=9606 GN=CLC PE=1 SV=3 |
